# Stimuli-Responsive Colloidal Gate for Active Modulation of Fluid Flow in Packed Beds

**DOI:** 10.1021/acs.langmuir.5c03082

**Published:** 2025-09-16

**Authors:** Gideon Onuh, Ronit Bitton, Oz M. Gazit, Ofer Manor

**Affiliations:** † The Wolfson Department of Chemical Engineering, Technion - Israel Institute of Technology, Haifa 3200000, Israel; ‡ The Department of Chemical Engineering, 26732 Ben-Gurion University of the Negev, Beer Sheva 84105, Israel

## Abstract

We present pH-responsive colloidal paste based on brushes of poly­(acrylic acid) (PAA) grafted on silica microparticles (∼4 μm) that actively modulates bulk fluid flow in packed bed columns of the colloid paste. pH-dependent charge and conformational changes of the PAA brushes in aqueous 10 mM NaNO_3_ solutions (pH 2–10) give an 8-fold change in flow rate (0.04–0.32 mL/min) through dynamic particle rearrangement in the paste, offering potential for flow rate modulation by pH. The colloid paste is a onsive “colloidal flow gate” that actively changes its permeability based on the aqueous solution acidity. We characterize the colloidal paste permeability by measuring pH variations of the flow rate through the paste.

## Graphical abstract

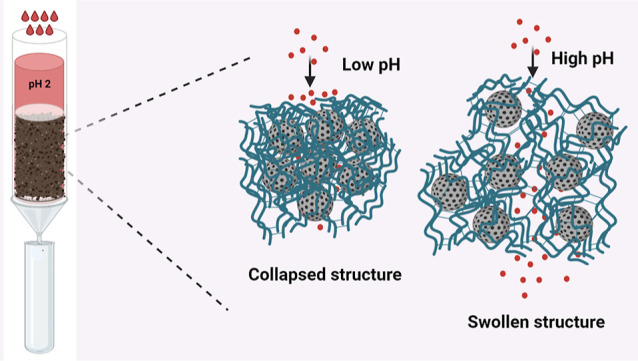

## 1 Introduction

Nature provides insightful models for designing stimuli-responsive functional materials. A notable example arises from clay minerals, which exhibit pH-tunable swelling and shrinkage of their interlayer galleries, permitting dynamic control of permeability and mechanical properties in response to their protonation state.^1,2^ This behavior, among other stimuli-responsive systems in nature, such as ion channels in cell membranes or plant stomata,^3,4^ inspires the design of synthetic materials with tunable transport properties. Mimicking such environmental behavior could produce new engineered architectures with controlled delivery,^5,6^ separations,^7–9^ sensing,^10^ and actuation^11–13^ applications.

Beyond particulates, pH-responsive transport has been extensively studied in porous membranes. The membranes offer analogous control over fluid flux and molecular release. For instance, polyelectrolyte multilayer membranes exhibit pH-dependent swelling to modulate water permeability,^14^ while hydrogel-based membranes enable pH-triggered drug release through conformational changes in polymer networks.^15,16^ These systems leverage surface charge and porosity variations to achieve stimuli-responsive behavior.

Control over liquid flow in porous beds via adjustable porosity has been further explored in smart porous materials, particularly through polymer-modified colloidal systems and membranes.^17–23^ Notable prior work includes pH-responsive colloidal membranes made from silica or gold nanospheres modified with poly­(methacrylic acid) (PMAA), which control ion and molecule transport via pH-driven polymer conformation changes,^24,25^ and in-column polymer growth on silica for stable chromatography columns.^20^

Building on these advances, our study introduces a pH-responsive packed bed system using silica microparticles (≈4 μm) functionalized by grafted poly­(acrylic acid) (PAA) brushes (PAA-Si). Our study utilizes pH-responsive silica microparticles functionalized with PAA to modulate fluid flow in packed bed columns, achieving dynamic control over permeability through colloidal interactions, offering a novel approach to stimuli-responsive transport. The transport of fluid through a particulate assembly is profoundly influenced by surface (intermolecular) forces and the resulting changes in the spatial organization of the particles in the assembly.^26–28^ Colloidal interactions, balancing van der Waals, electrostatic, and steric contributions, guide nanoparticle packing and porosity within coatings.^29,30^ Variations in Hamaker constants, surface potentials, and steric layer thicknesses recalibrate packing densities and interparticle dimensions.^31,32^ Our analysis has similarities to pervious membrane work. However, the dynamic rearrangement of particles in particulate packed beds, as in our study, provides a unique approach to tuning permeability through colloidal interactions, distinct from the fixed pore structures of membranes.

## 2 Experimental Section

### 2.1 Surface Modification of Silica Support

The stimuli-responsive silica microparticles functionalized with poly­(acrylic acid) (PAA) brushes, shown in Figure 2a, were synthesized via modification of methods previously described.^33,34^

#### 2.1.1 Calcination of Silica Support

Silica microparticles (6 μm, ACEMATT OK 412, CAS-No. 112926-00-8) were purified via acid washing and calcination. Three grams of the silica powder was dispersed in 1 M hydrochloric acid (ACS reagent, Sigma-Aldrich, 37% (w/v)), followed by heat treatment ramping at 10 °C/min to 800 °C in an air atmosphere and an isothermal dwell at this temperature for 12 h using Furnace KR1, Ascon Technologic. This calcination process served to remove organic contaminants from the silica surface through acid-catalyzed pyrolysis, yielding pure microparticles for subsequent functionalization experiments.

#### 2.1.2 Grafting of (3-aminopropyl)­triethoxysilane (APTES)

Surface silanols on the purified silica microparticles were hydrolyzed to facilitate covalent grafting. One gram of the calcined silica powder was refluxed in distilled H_2_O at 100 °C for 12 h to induce complete hydrolysis of residual siloxane species. Solvent exchange was then performed via sequential centrifugation and resuspension in anhydrous ethanol (Bio-Lab, AR dehydrated) (3×), tetrahydrofuran (Bio-Lab) (2×), and toluene (Bio-Lab) to remove trace water before silanization.

Control over grafting density was achieved through systematic variation of the aminopropyltriethoxysilane (APTES) concentration (1, 5, 8, and 10% v/v, Merck, CAS: 919-30-2, 99%) in 40 mL of dry toluene (Bio-Lab, AR-b anhydrous, cat no:002015052100, 99.8%) in a fume hood under reflux in air at controlled temperature (25 °C) and pressure, with magnetic stirring (500 rpm, 12 h). The reflux setup with anhydrous toluene minimized moisture-induced hydrolysis, ensuring uniform APTES grafting. The APTES grafting process involves hydrolysis of ethoxy groups to form silanol groups, which undergo multicondensation with surface silanols on silica and neighboring APTES molecules, forming a dense, multilayered amine-functionalized layer.^35^ This “carpet” of amine functionalities, characterized by acid–base titration (Table 1, 1.65–2.74 molecules/nm^2^), zeta potential measurements (Figure 2d), and thermogravimetric analysis (Figure 2e), provides a high density of reactive sites for PAA conjugation. The progressive APTES hydrolysis and co–condensation reactions afforded amine-functionalized particles, code-named as low (1% APTES-Si), moderate (5% APTES-Si) and high (10% APTES-Si) samples. Thorough washing with ethanol ensured the removal of physisorbed and unreacted APTES before poly­(acrylic acid) conjugation.

**Table 1:** APTES Loading Density on Silica Particles Calculated from Acid–Base Back Titration

APTES	SiO_2_ surface area	APTES (ρ)	APTES
(%)	[m^2^g^–1^]	[μmolg^–1^]	[molecules nm^–2^]
1	5.4	1.5	1.65
5		1.9	2.08
10		2.5	2.74

#### 2.1.3 Poly­(arylic) Acid Conjugation

Amine-terminated silica microparticles synthesized previously were functionalized with poly­(acrylic acid) (PAA) via carbodiimide chemistry. Briefly, 30 mg of PAA (Sigma-Aldrich, *M*_W_: 100,000 Da) was dissolved in phosphate-buffered saline (PBS, pH 7.4, Sigma-Aldrich) and activated by addition of 0.1 M 1-ethyl-3-(3-(dimethylamino)­propyl) carbodiimide (EDC, TCI product, CAS: 25952-53-8, ≻ 98.0%) and 0.1 M *N*-hydroxysuccinimide (*N*HS, Acros Organic, CAS: 6066-82-6, ≻ 98.0%). The reaction mixture was stirred at room temperature for 3 h to form an *N*HS-ester intermediate on the carboxylic acid moieties. The activated PAA solution was then slowly added dropwise to the amine-functionalized silica slurry (dispersed in PBS) under constant stirring at 500 rpm. Amine-NHS coupling was facilitated by refluxing the dispersion at 100 °C for 6 h. The PAA-grafted particles were isolated by centrifuging at 5000 rpm using Hermle Z 326 K Centrifuge for 5 min, followed by sequential washing with excess PBS and ethanol to remove physisorbed and unreacted PAA. Finally, the particles were dried under vacuum at 110 °C for 1 h to ensure the removal of residual solvent.

### 2.2 Characterization Methods

The APTES grafting densities on the functionalized silica microparticles were quantified via acid–base titration, following procedures detailed in the Supporting Information (Figure S2). Briefly, the amino groups were titrated against hydrochloric acid and the consumption of HCl equivalents was used to determine the surface-bound amine concentrations. Furthermore, the chemical and physical properties of the PAA-Si particles were characterized using a suite of techniques. Diffuse reflectance infrared Fourier transform (DRIFT) spectroscopy for spectra of modified surfaces, small-angle X-ray scattering (SAXS) due to polymer brush density, thermogravimetric analysis (TGA) for organic content of the silica after thermal decomposition, dynamic light scattering (DLS) of changes to particle hydrodynamic sizes post-functionalization, and zeta potential measurements were performed with specific instrument settings and software, as detailed in Supplementary Table S1.

### 2.3 pH Responsive Nature of Silica Particle-Grafted PAA

To systematically investigate the pH-responsive behavior, freshly prepared suspensions of the surface-modified silica microparticles were utilized for each characterization measurement.^7^ Particle concentration was fixed at 1 mg/mL while pH was modulated from acidic to basic conditions via incremental addition of calculated volumes of HCl (Bio-Lab) and NaOH (Bio-Lab). To maintain a constant ionic strength throughout pH adjustment, 1 mL of a 10 mM NaNO_3_ solution was added. DLS and zeta potential analyses were carried out in dilute suspensions of a concentration of 1 mg/mL in pH-adjusted 10 mM NaNO_3_ solutions (pH 2–10). The impact of varying pH on both zeta potential and hydrodynamic diameter was then assessed in situ using a Malvern Zetasizer nano instrument. Additionally, particulate aggregation processes were simultaneously monitored under an optical microscope (Eclipse, Ni-E, Nikon High-Technologies Corporation, Japan), and aggregate sizes were estimated from captured images using ImageJ software.

#### 2.3.1 Reversibility of pH-Responsive Particle Behavior

To assess the instantaneous pH-driven reversibility, a 5 mg/mL suspension of *h*-PAA-Si was prepared at pH 4 and equilibrated for 30 min. After sedimentation, the supernatant was decanted, and a portion of the sediment was diluted to 1 mg/mL for DLS analysis to determine aggregate sizes. The remaining sediment was resuspended in a pH 9 solution, vigorously agitated, and allowed to settle for 30 min before repeating the measurement. The pH alternation (4 to 9 and back) was cycled multiple times to evaluate rapid responsiveness.

For time scale-dependent reversibility, separate suspensions were incubated overnight (≈12 h) at pH 3 and pH 9. Aggregate sizes were then measured via DLS, and 5 μL aliquots were drop cast onto glass slides for in situ optical microscopy of aggregates. Subsequently, pH was alternated between 3 to 9 with 30 min equilibration intervals. All measurements were performed in triplicate to ensure reproducibility and reliability of collected data.

### 2.4 Colloidal Gate Model Setup and Flow Rate Measurement

A flow gate device was designed using a standard 3 mL disposable polyethene syringe (Norm-Ject, D-78532, Germany) as the fixed-bed column, following the previous design by Khaskia et al.^7^ The column was packed as follows: at the base, two 25 μm pore-size Whatman filter paper discs with a dialysis membrane in between were layered to prevent particulate leakage. Next, the stimuli-responsive modified silica samples (50 mg total weight) were packed to a 4 mm bed height. Finally, two additional 25 μm filter paper discs were layered on top to prevent particle displacement during liquid flow. Buffer solutions across the pH range of 2–10 were sequentially introduced to the column inlet to hydrate the packed bed at a constant hydrostatic pressure, followed by precise monitoring of effluent volumes collected over time. Critical to maintaining a consistent pressure head, the liquid volume above the packed bed was kept constant at 2.5 mL for all measurements. Flow experiments were conducted under isocratic conditions and repeated in triplicate to ensure the repeatability of results. Flow rate was calculated from the slope of cumulative effluent volume versus time (measured over 1 h) using a digital stopwatch at a precision of ≃ 0.1 s, with triplicate measurements.

## 3 Results and Discussion

In our experiments, we study the flow rate through particulate beds of modified silica materials prepared by coupling poly­(acrylic acid) (PAA) to (3-Aminopropyl)­tiethoxysilane (APTES) grafted on the silica surface at low (1%), moderate (5%), and high (10%) APTES densities; see Figure 1. We use the corresponding terms: *l*-PAA-Si, *m*-PAA-Si and *h*-PAA-Si, respectively, to indicate the different particle surface concentrations of PAA. Protonation and deprotonation of the surface-bound PAA are toggled by systematically varying the buffer pH. Below the p*K*_a_ of 4.26,^36^ carboxylic acid groups are predominantly protonated, instigating interparticle association through electrostatic interactions and hydrogen bonding.^37^ This triggers bed compaction, which arrests the flow. In contrast, at high pH, the carboxylate moieties deprotonate and thus extend into the solvent, repelling neighboring particles, dispersing the colloid, and opening the way for fluid flow.

**Figure 1:**
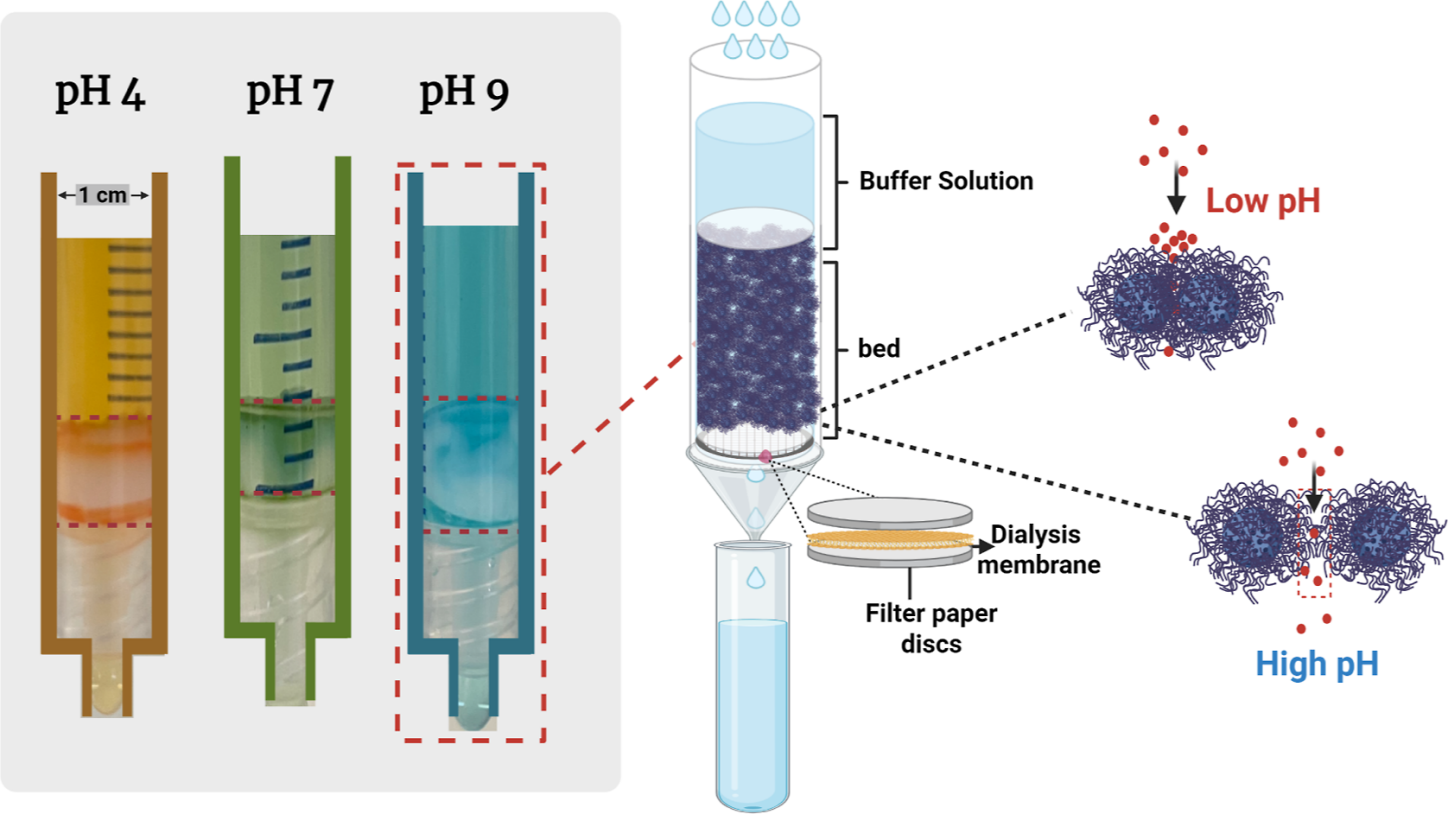
Schematic illustration of particulate packed beds in columns and the interaction between particles therein at different pH levels.

### 3.1 Materials Characterization

The successful functionalization and modification of the materials used in this study were characterized as follows: Shown in the supplementary scheme (Figure S1), we conjugate poly­(acrylic acid) (PAA) to aminopropyltriethoxysilane (APTES)-functionalized silica microparticles through amidation linking of amines and activated carboxyl groups.^34^ The density of surface amine sites on APTES-functionalized silica particles was determined by acid–base titration, where particles were titrated with 0.1 M HCl to protonate amine groups, followed by back-titration with 0.1 M NaOH; see supplementary file, Figure S2. The consumed HCl content divided by the total particle surface area (≈4 μm diameter, calculated from particle mass and geometry) was converted in our analysis to the reacting amine density (see Table 1). In Table 1, we quantify the APTES grafting densities achieved by systematically varying the sol–gel reaction concentrations to 1%, 5%, and 10% APTES, which we characterized to be 1.5, 1.9, and 2.5 μmol APTES per gram of silica, respectively, using acid–base titration.^38^ Corresponding areal densities were computed to be 1.65, 2.08, and 2.74 APTES molecules per nm^2^.^39^ PAA chains (*M*_w_ ≃ 100 kDa, Sigma-Aldrich) were attached to APTES sites via a “grafting-onto” method using EDC/NHS coupling. Due to steric hindrance, not all APTES sites were functionalized with PAA, with thermogravimetric analysis (TGA, Supplementary Figure S3) estimating a PAA grafting density of 0.5–1.0 chains/nm^2^ for 1–10% APTES samples. This density may moderately form a dense brush-like structure, sufficient to drive pH-dependent particle interactions, though lower than densities achievable via “grafting-from” methods.^40^ The intergrafting distance for APTES (≈1 nm for 10% APTES) was calculated assuming uniform distribution, although PAA spacing is likely higher due to incomplete functionalization.

Diffuse reflectance infrared Fourier transform (DRIFT) spectroscopic analysis in Figure 2b provides insights into surface functionalization. The calcined, hydrolyzed silica support exhibits intense peaks attributable to siloxane bonds (1100 cm^–1^) and broad silanol features (3400 cm^–1^). Grafting (APTES) at 5%, 8%, and 10% concentrations introduces characteristic N–H stretches (3300 cm^–1^), validating amine incorporation onto silica. Notably, the C–H stretching vibrations (2800–2900 cm^–1^) intensify with increasing APTES density during the reactions. Upon conjugating poly­(acrylic acid) (PAA, 30 mg) to the amine-functionalized surfaces, amide bond formation was evidenced by emerging N–H bending (1400 cm^–1^) and C=O stretches (1600 cm^–1^). The broad O–H peak (3500 cm^–1^) associated with carboxylic acids was also enhanced for polymer-bound samples.

**Figure 2:**
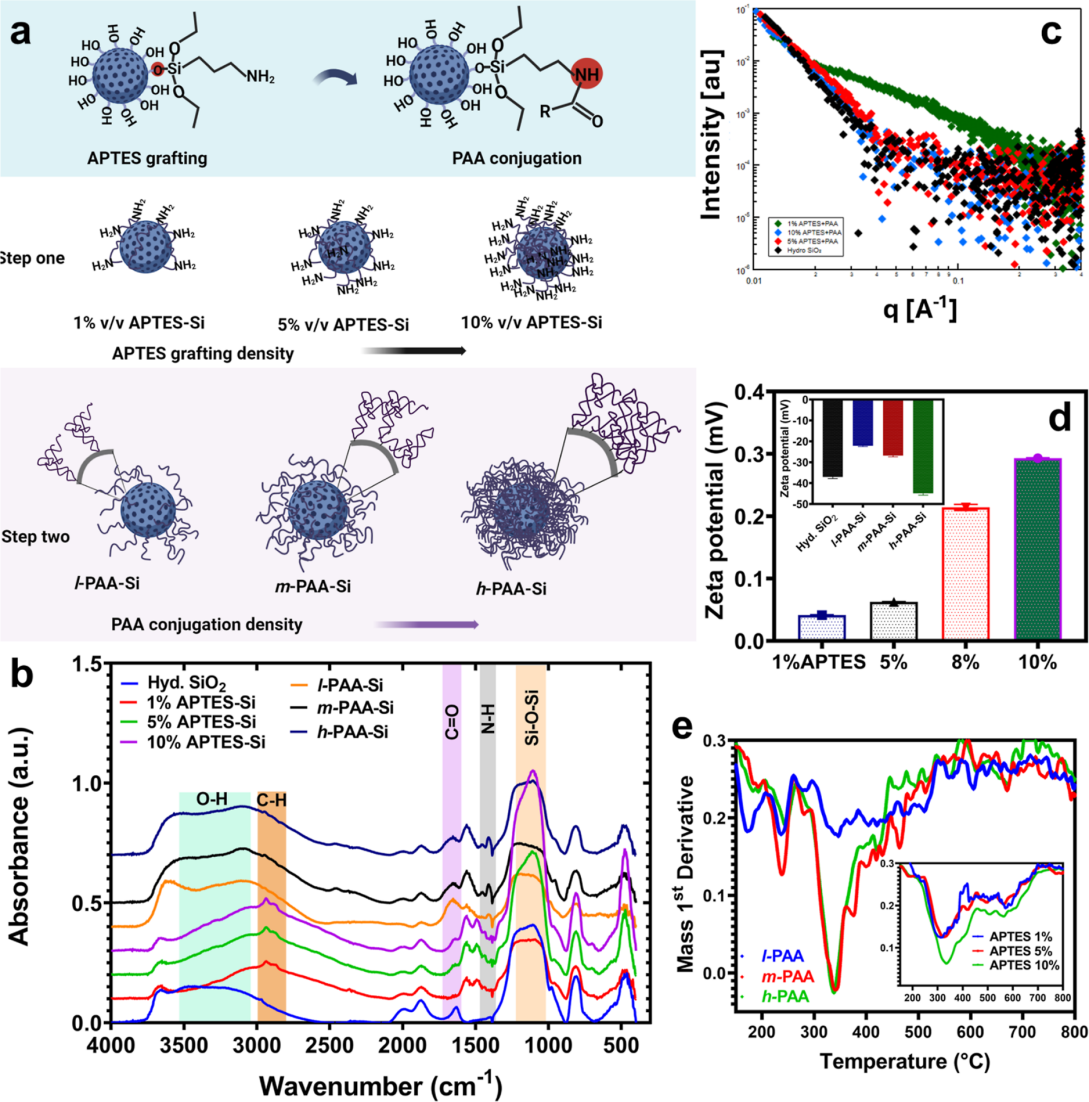
(a) Schematic illustration of stepwise functionalization of silica microparticles (≈4 μm diameter) with progressive surface modification. Aminopropyltriethoxysilane (APTES) grafting at 1, 5, and 10% (v/v) concentrations covalently introduces primary amine-terminating moieties, with density quantified via acid–base back-titration (Table 1). (b) DRIFTS spectra for concentration-dependent APTES grafting and subsequent PAA conjugation onto silica microparticles. (c) SAXS measurement of unmodified silica and *l*-PAA-Si, *m*-PAA-Si, and *h*-PAA-Si (d) Zeta potential measurements in 5 mM NaNO_3_ solution showing variation in silica microparticles’ surface charge density after stepwise surface modification with APTES and PAA-Si. (e) DTG analyses of the organic content of APTES and PAA-Si samples after thermal decomposition.

SAXS measurements of 1, 5, and *h*-PAA-Si samples in aqueous solutions (pH = 9), Figure 2c show that only the particle of *l*-PAA-Si exhibits a different scattering pattern than that of the unmodified silica. It should be noted that within the measured q-range, it is anticipated that only the characteristics of the grafted PAA will affect the scattering curve. A simplistic calculation of the available surface for each grafted PAA chain indicates that the average distance between two grafting points is ≈10 nm for *l*-PAA-Si, ≈2 nm for *m*-PAA-Si, and ≈1 nm for *h*-PAA-Si. Only the 10 nm distance is larger than the expected R_*g*_ of 100 kDa PAA.^41^ Therefore, in the case of 5% and *h*-PAA-Si, the grafted chains organize into a dense layer in which the conformation of a single chain is not distinguishable, and the entire PAA layer can be assumed as an integral part of the silica particle.

Zeta potential measurements (Figure 2d further validate stepwise surface functionalization by quantifying evolving charge densities. As-prepared silica microparticles exhibit a ζ-potential of −37 mV, consistent with deprotonated silanols.^42^ Following aminopropylsilane grafting, surfaces became increasingly positively charged, with values of +0.05, +0.08, +0.23, and +0.3 mV attained through 1%, 5%, 8%, and 10% APTES concentrations, respectively. Subsequent PAA conjugation imparts renewed negative character, shifting potentials to −22, −28, and −44 mV for the *l*-PAA-Si, *m*-PAA-Si, and *h*-PAA-Si systems, respectively.

Thermogravimetric analysis profiles (Figure 2e and Supplementary Figure S3) provide insights into changes in the chemical constitution of (APTES-grafted and PAA-conjugated) samples imparted by stepwise surface functionalization. For PAA-Si samples, the emergence of distinctive weight loss peaks at 190 and 210 °C, absent from primary APTES controls, confirms successful polymer attachment. Also, we observed an increase in mass loss at 340 °C associated with PAA decomposition.

### 3.2 pH-Dependent Aggregation and Reversibility

In Figure 3, we quantitatively track the aggregate sizes (associated with *h*-PAA-Si materials) as a function of pH. The aggregates disperse at high pH levels, as expected based on the deprotonation of the poly­(acrylic acid) (PAA) corona. Below the p*K*_a_, larger-size aggregates form due to protonation-instigated interparticle association. Notably, in suspensions that satisfy a pH level of 2–4, the particles display substantial aggregate growth to ≈57–59 μm, which is correlated to the reduced surface charge, measured by zeta potential analysis (see Figure 3a). A maximum agglomerate size of ≈59 μm is measured at pH 3, which coincides with the point of zero charge (PZC) of the PAA-functionalized silica. Here, van der Waals attractions outweigh electrosteric repulsions imparted by protonation/deprotonation of tethered PAA chains, which facilitates coagulation.

**Figure 3:**
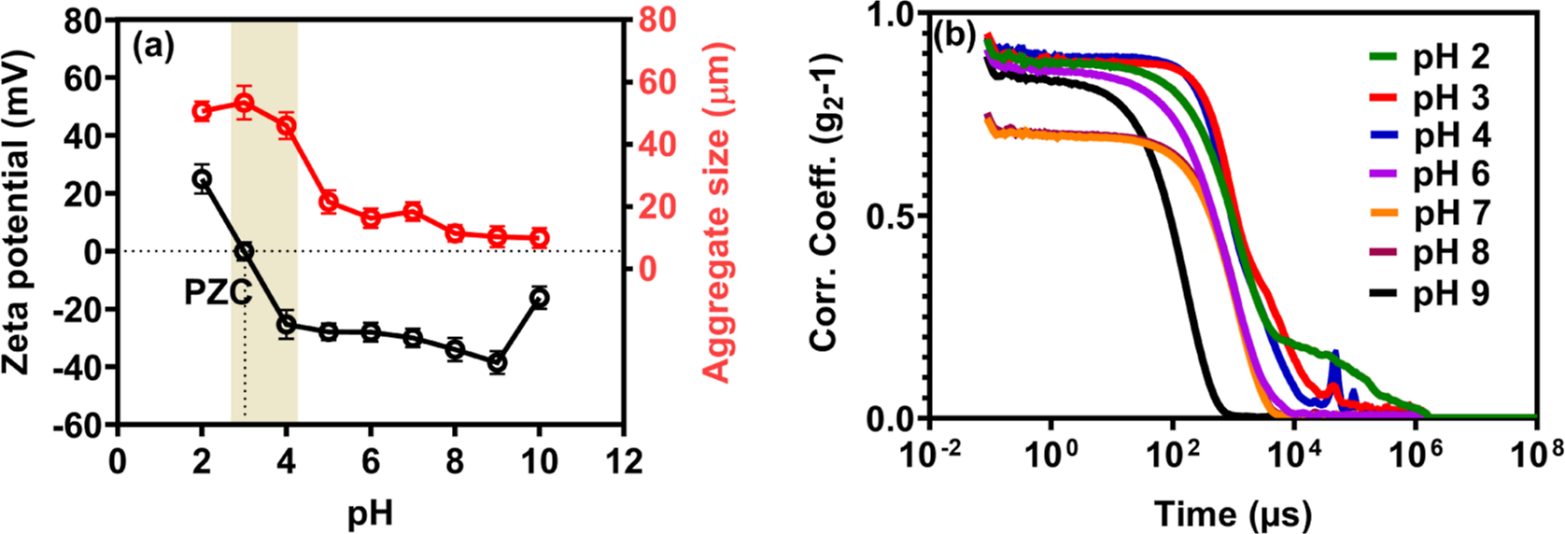
(a) pH variations of the zeta potential on *h*-PAA-Si particles and aggregate size measurement using DLS (b) corresponding autocorrelation function obtained from DLS measurement showing delay time in the dispersion. Error bars in (a) indicate standard deviation calculated using triplicate measurements.

Dynamic Light Scattering (DLS) provides a measure of the intensity fluctuations over time through the second-order correlation function, g^2^(τ), where τ denotes time delay.^43^ As observed from the autocorrelation function (Figure 3b), larger and slower particles maintain a high correlation for longer time scales, while smaller particles exemplify faster Brownian motion, which renders the autocorrelation function decay at short time scales.^43,44^ For example, at pH level 3, where maximum aggregate dimensions are observed, the second-order correlation function decayed over a long time scale, which is consistent with large-sized aggregates. In contrast, at a pH level of 9, a condition favoring particle dispersion, the second-order correlation function decayed over a short time scale. The lower initial amplitudes of autocorrelation functions at pH 7 and 8 in Figure 3b, may reduced scattering intensity due to partial PAA chain extension near the p*K*_a_ (≈4.5–5.5), which alters the refractive index contrast and aggregation state.^45^

We examine the reversibility of the *h*-PAA-Si particle system by monitoring particle coagulation and dispersion under pH cycles, using DLS and optical microscopy. Shown in Figure 4a, our particle system exhibits robust reversibility between pH 4 and pH 9. We alternate the suspension pH between 4 and 9 over a 5 h period, allowing 30 min of equilibration at each pH level before measuring the hydrodynamic diameter (D_*h*_) of aggregates via DLS. At pH 4, the characteristic diffusion time of the aggregates was ≈3000 μs and is driven by protonation-induced coagulation. At pH 9, the characteristic diffusion time decreases significantly to ≈300 μs, reflecting dispersion of aggregates, which is driven by deprotonation and enhanced electrostatic repulsion; see Figure 4a,b. The characteristic diffusion time (τ) was calculated from the DLS autocorrelation function using the relation $\tau =\frac{1}{Dq^{2}}$ , where *D* is the diffusion coefficient derived from the Stokes–Einstein equation $D=\frac{k_{B}T}{3\pi \eta d_{h}}$ (with k_B_ as the Boltzmann constant, *T* = 298 K, η as the viscosity of water, and *d*_*h*_ as the hydrodynamic diameter), and $q=\frac{4\pi n}{\lambda }\sin\left(\theta /2\right)$ is the scattering vector (with *n* = 1.33, λ = 633 nm, θ = 173°); see detailed calculations in the supplementary file, section 2.0. These changes reflect the collapse and extension of PAA chains due to protonation, which results in electrosteric repulsion.

**Figure 4:**
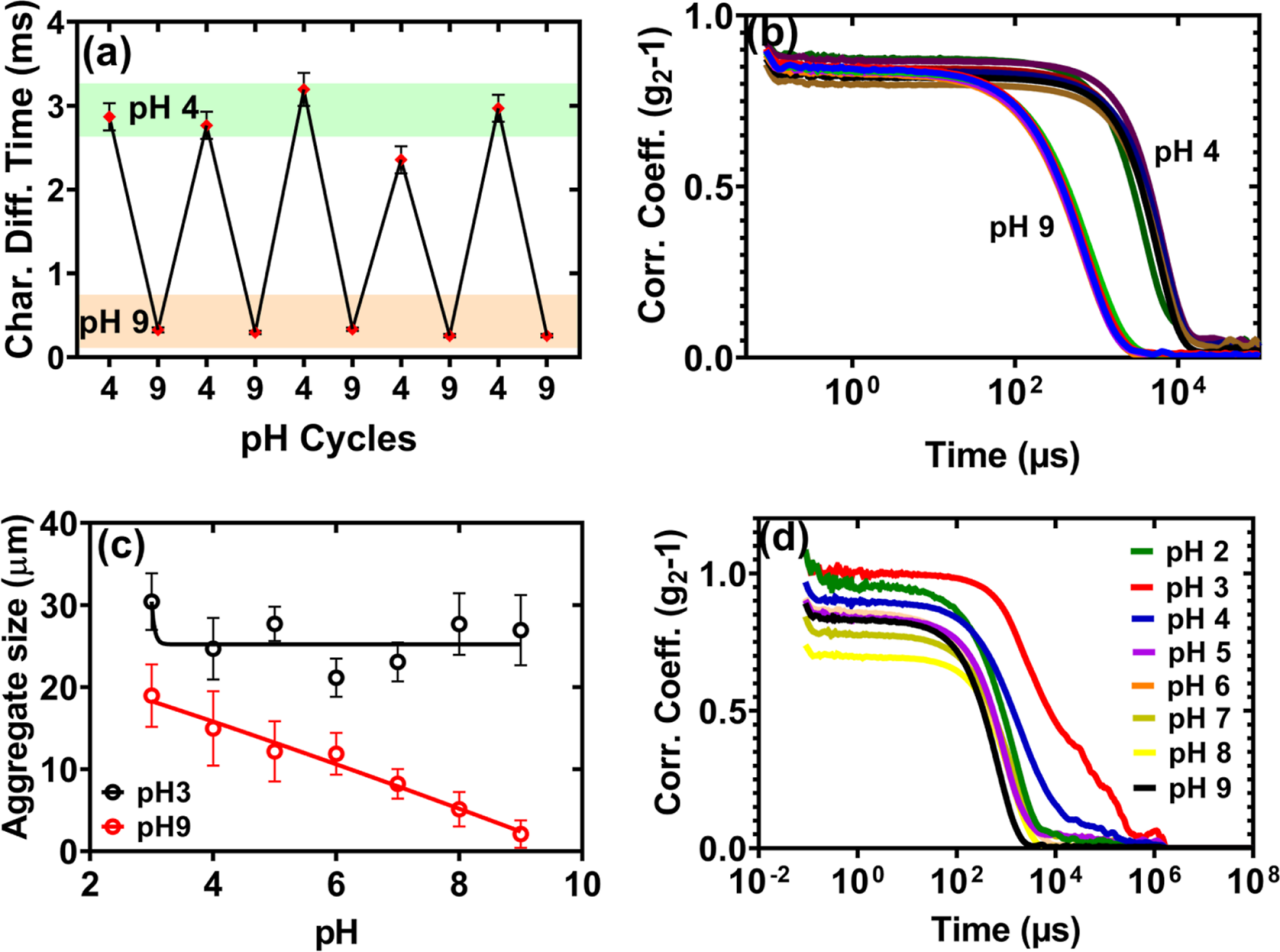
Reversibility of pH responsiveness of *h*-PAA-Si sample: (a) characteristic diffusion time of aggregates measured using DLS measurements by alternating the pH of the suspension between 4 and 9. (b) Corresponding autocorrelation function for Figure 4a between pH 4 and 9. (c) pH variations of direct aggregate size measurement using light microscopy images as a function of pH (by incubating the suspension at pH 3 and 9 for ≈12 h and reversibility within 30 min equilibration time). Error bars indicate standard deviation from triplicate measurements. The maximum errors after 24 h equilibration time was ≈5% (d) corresponding autocorrelation function for Figure 4c at pH 9; see Figure S5 (supplementary file) for reversibility at pH 3.

We further left the suspensions overnight (≈12 h) at pH 3 and pH 9, and monitored the aggregate size via optical microscopy (Figure 4c) and DLS, Figure 4d, also, see Figure S5 (supplementary file). At pH 3, aggregates grew to about 27 μm, a significant increase from the size of detached particles. This is van der Waals-driven coagulation near the point of zero charge (PZC) of the suspension. In contrast, at pH 9, particles remained dispersed at ≈ 4 μm. This is consistent with the presence of electrosteric and steric repulsion between polymer brushes on different particles. In this case, upon altering the pH level from 3 to 9 with a 30 min equilibration period, the large aggregates formed at pH 3 exhibited irreversible compaction; see (Figures 4c and S5. In contrast, dispersed particles at pH 9 aggregated upon decreasing the pH of the suspension (Figure 4c). Sizable aggregates grew approximately 20-fold relative to the original particle size. Correspondingly, autocorrelation delay times increased progressively (Figure 4d), confirming the observed particulate sizes. The system’s irreversibility following an overnight exposure to pH 3 level acidity may result from strong van der Waals interactions between particles due to negligible polymer effect at the PZC of the materials. In addition, extended aggregation at pH 3 may lead to minor PAA chain entanglement, potentially slowing dispersion kinetics at high pH levels.

To visualize the state of the particulate dispersion, in Figure 5, we present optical microscopy imagery tracking pH-dependent particulate deposition and aggregation. Observations are made in situ in the suspension (see Figure S7) and immediately following solvent evaporation. In the latter case, capillary stresses further push the particles together during the final stages of the solvent evaporation, albeit clear differences are observed between different particle specimens according to the original pH level in the solvent. Densely clustered aggregates dominantly populate the substrate at pH levels of 3, 4, and 10. At pH levels of 3 and 4, the relatively dense particulate deposition corresponds to the low surface charge of the particles in the solvent (given in terms of zeta potential in Figure 3a). At a pH level of 10, we further observe dense particulate deposition. The slight variation in zeta potential magnitude observed between pH 9 and 10 may be attributed to the presence of counterion (Na^+^), which are introduced during pH adjustment of the solutions using NaOH. However, this variation may not result in significant aggregation, as dispersion remains predominant, which is evidenced by the consistent flow rate behavior observed in Figures 6a and 7. The microscopic images qualitatively mirror quantitative light scattering data, showing pronounced aggregation and surface particulate coverage under conditions favoring electrostatically/sterically mediated coagulation.

**Figure 5:**
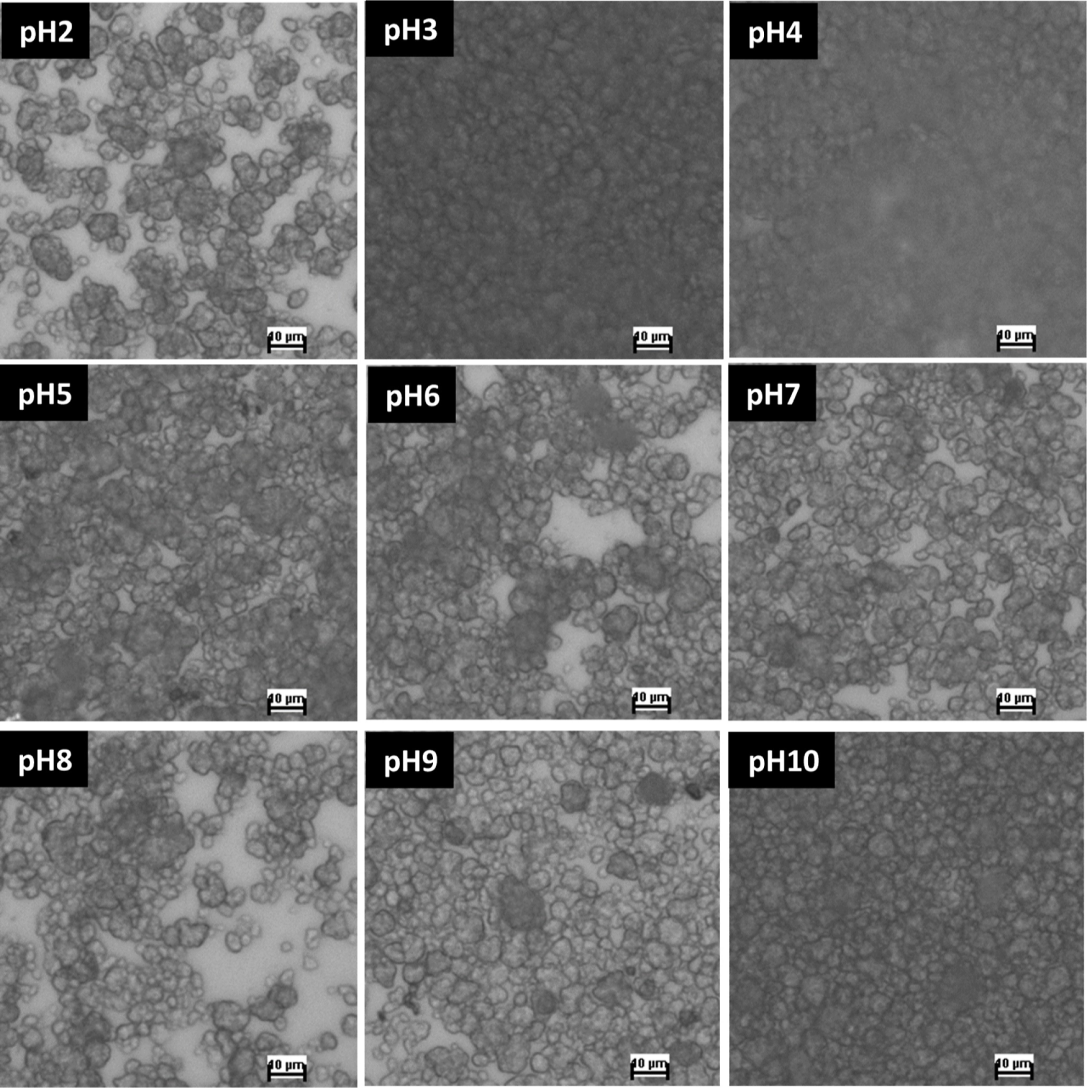
In situ aggregate deposit images from 5 μL of *h*-PAA-Si particulate suspension under light microscopy, showing the effect of pH on the rate of particle coagulation and adsorption to the solid substrate.

**Figure 6:**
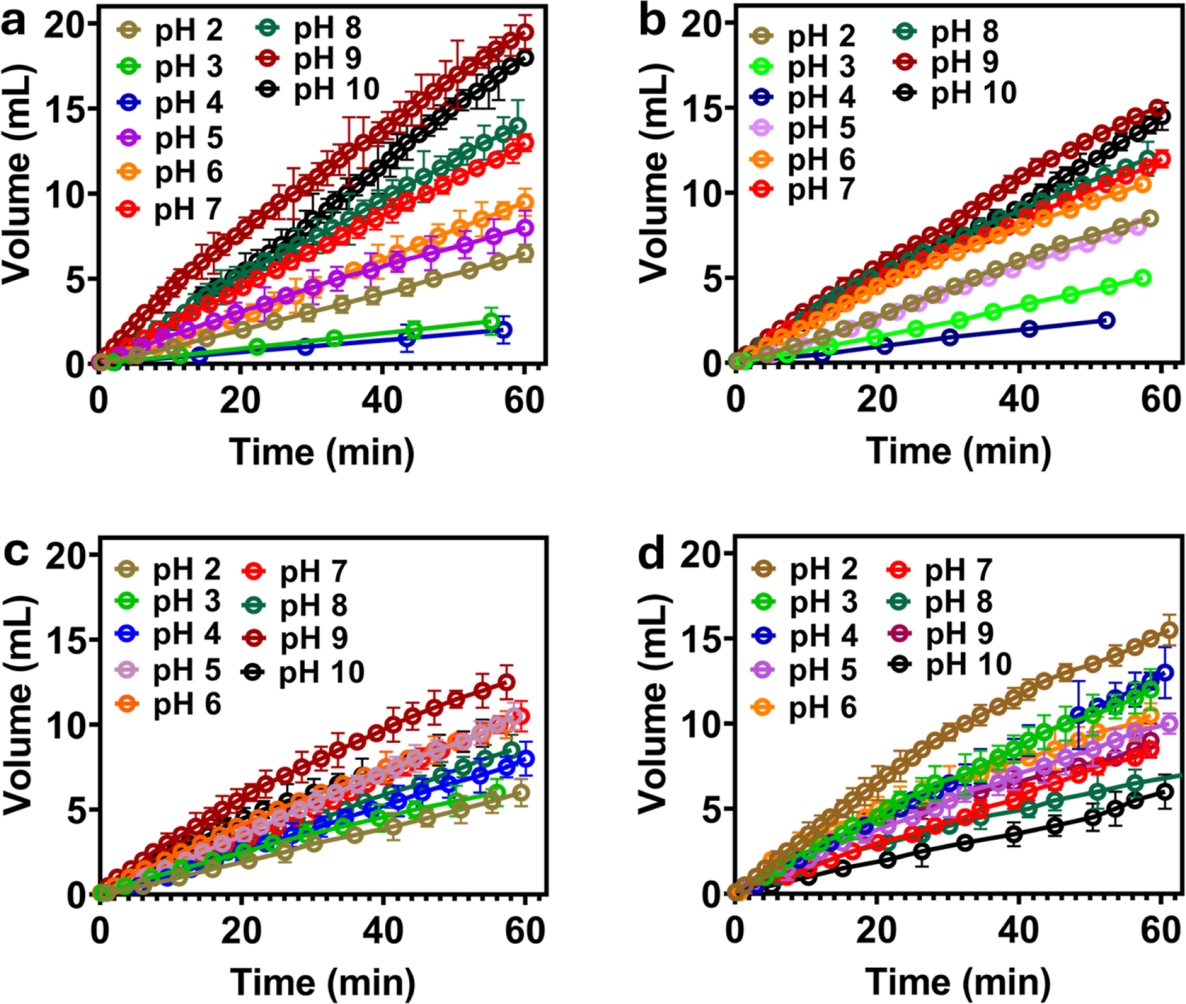
Time variation of fluid volume passing through the particulate packed bed for (a) *h*-PAA-Si functionalized particles, (b) *m*-PAA-Si functionalized particles, (c) *l*-PAA-Si functionalized particles, and (d) nonfunctionalized Si (silica) particles. The measurements were conducted under identical conditions using the same flow cell assembly and buffer solutions.

**Figure 7:**
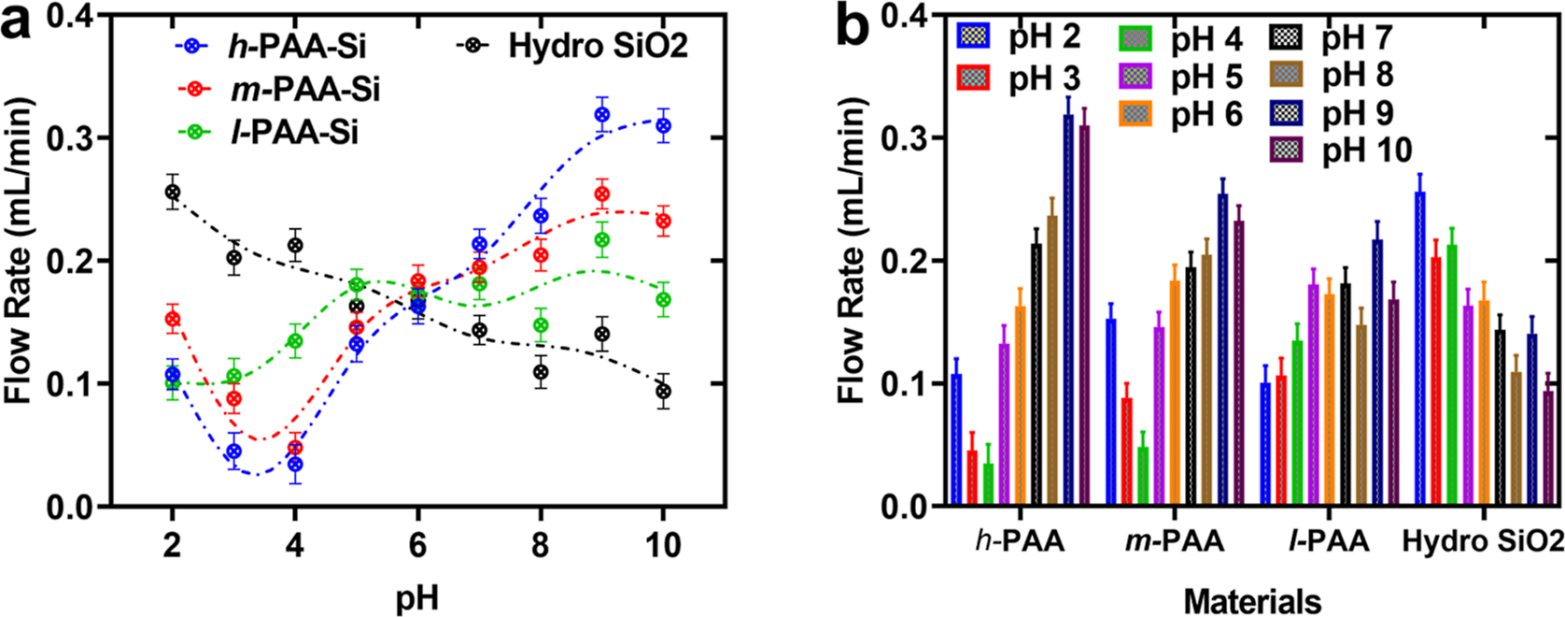
(a) Flow rates vs pH showing liquid flow behavior across varied polymer density. (b) Plots of *l*-PAA-Si, *m*-PAA-Si, and *h*-PAA-Si functionalized particles and nonfunctionalized Si (silica) particles vs pH. The dash lines are added to guide the eye.

### 3.3 Permeability and Transport Modulation

To evaluate the functional application of our surface-engineered materials, we quantify liquid permeability through packed columns using the setup detailed in Figure 1. We recognize that surface charge densities and colloidal interactions are sensitively coupled to both pH and ionic strength.^46^ We add 10 mM NaNO_3_ to the buffer solution to decouple the two effects. Thus, we buffer the background ionic strength, since varying pH induces counterion dissociation that would otherwise alter solution composition. By fixing ionic strength across the tested pH range, surface properties are directly correlated to the protonation state, and thus, we may attribute observed behaviors solely to changes in the solvent pH.

As shown in Figure 6, the flow rate increases monotonically with increasing pH for the PAA-Si bed. The sample modified with PAA onto 10%(v/v) aminosilane-functionalized silica (*h*-PAA-Si) (Figure 6a) exhibits a 10-fold increase in liquid permeation between pH 4 and pH 9 compared to the cases of *l*-PAA-Si & *m*-PAA-Si materials. The lowest flow rate is observed at pH 4, which is near the point of zero charge (PZC) of the particles, where we expect electrosteric contributions to bed compaction and permeability to be negligible. Under these conditions, particle–particle interactions are predominantly governed by van der Waals forces in the absence of significant electrostatic or steric repulsion. Moreover, prior investigations demonstrated that attractive van der Waals forces can strongly influence bed compaction by facilitating aggregation and tighter packing arrangements between particulates.^29^ This compaction is shown here to modify bed porosity, tortuosity, and connectivity in a manner that hinders liquid transport therein.

We find that the *h*-PAA-Si material outperforms the *l*-PAA-Si and *m*-PAA-Si variants, which show reduced modulation (Figure 6b,c). Detailed characterization of our colloid suspension below suggests that higher APTES concentrations enhance PAA conjugation through increased covalent linkages between carboxylic acid groups and amine sites. This yields a denser polymer brush morphology in the *h*-PAA-Si particle compared to the *l*-PAA-Si and *m*-PAA-Si particles.^47^ We monitor the flow for 1 h to ensure equilibrium between liquid and surface acid/base groups to fully isolate the effect of deprotonation and protonation of PAA brushes at varied pH on bed porosity and flow through the column. Higher APTES concentrations, yielding denser PAA layers, promote cohesive aggregation at low pH, restricting flow, and pronounced dispersion at high pH, amplifying transport modulation. Conversely, lower APTES loadings (Figure 6c) result in reduced PAA conjugation, leading to weaker interparticle interactions and a diminished flow-modulation property due to fewer polymer chains available for pH-dependent conformational changes.

The flow measurements through the packed beds align with prior zeta potential characterizations of the PAA-conjugated silica particles as a function of pH. Specifically, electrophoretic analyses of *h*-PAA-Si reveal a zeta potential increase from +21 mV to −43 mV across a pH level range of 2 to 9, with a mild decline to −22 mV at pH 10. This shift confirms enhanced deprotonation of surface PAA/silica groups with increased pH, augmenting the overall charge density and hydrophilicity relatively to lower pH conditions. The correlated changes in electrokinetic potential validate the presence of amplified electrostatic repulsion and extended steric dimensions imposed on neighboring particles under increasingly ionized states. This ionization-driven expansion of the PAA chains increases the polymer radius of gyration, capable of exerting augmented electrosteric forces between particles,^48^ which mitigate particle packing efficiency.

In contrast to the pronounced pH-responsive flow modulation observed in PAA-Si materials, where electrosteric repulsion drives enhanced permeability at high pH levels (Figure 6a–c), unmodified silica particles (Figure 6d), exhibit a divergent transport behavior. Zeta potential measurements of bare silica particles in Figure S6 (supplementary file) reveal an increase in negative surface charge density from −9 mV to −42 mV as we increase the pH from 2 to 10, respectively, which is consistent with silanol group deprotonation.^49^ Permeability assessments show a decrease in liquid flow through packed beds of unmodified silica at higher pH (Figure 6d). While increased surface charge might be expected to promote looser colloidal arrangements, competing surface forces and morphological factors likely consolidate the irregular silica assembly at elevated pH. Specifically, heterogeneity in particle size and shape distributions may induce packing inconsistencies that obstruct flow pathways.^50^ Moreover, hydrogen bonding and residual van der Waals interactions may outweigh electrostatic repulsions at high pH levels, enhancing interparticle cohesion.^51^ The classical Derjaguin–Landau–Verwey–Overbeek (DLVO) theory appears not to account for the permeability behavior in the case of our unmodified silica.

In Figure 7a,b, we summarize the flow rate across different polymeric systems. The *h*-PAA-Si material exhibits the most pronounced pH-responsive behavior, achieving a flow rate of 0.04 mL/min at pH 4 and a peak of 0.32 mL/min at pH 9, corresponding to an 8-fold modulation. The *m*-PAA-Si variant displays moderate pH tunability, with flow rates ranging from 0.05 mL/min at pH 4 to 0.21 mL/min at pH 9. The *l*-PAA-Si system shows minimal permeability variation, with flow rates between 0.10 mL/min at pH 2 and 0.18 mL/min at pH 9, underscoring the critical role of PAA density in modulating the particulate permeability.

Moreover, during PAA conjugation via EDC/NHS chemistry, the presence of multiple NHS-ester groups per PAA chain (*M*_W_: 100,000 Da) raises the possibility of multipoint grafting to surface amines, particularly at high APTES densities (e.g., 10%, ≈1 nm between sites). This could result in intraparticle cross-linking, constraining PAA chain flexibility, or, less likely, interparticle cross-linking, bridging adjacent particles. SAXS data (Figure 2c) suggest that *m*-PAA-Si and *h*-PAA-Si form dense, integral PAA layers, consistent with possible multipoint grafting. However, the robust reversibility of aggregation/dispersion (Figure 4) and significant flow modulation (Figure 7) indicate that any cross-linking does not significantly impair pH-responsive behavior. The high PAA density in the case of *h*-PAA-Si likely enhances electrosteric repulsion, which compensates for reduced chain flexibility.

In addition, we investigate the contribution of the particulate packing density and ionic strength to the *h*-PAA-Si particles within columns. Varying the packed mass of *h*-PAA-Si from 50 mg to 150 mg reduces the flow rate across all pH levels. This is consistent with Darcy’s law, which describes the relationship between the flow rate (*Q*) and the hydraulic head (*h*) through the equation *Q* = (*kA*/μ*L*)*h*, where *k* is the permeability of the particulate bed, *A* is the cross-sectional area, μ is the fluid viscosity, and *L* is the length of the bed along the main path of the flow.^52^ The flow rate can be further expressed as *Q* = (*kA*Δ*P*)/μ*L*, where Δ*P* is the pressure drop across the bed. As the bed height (*L*) increases, the hydraulic resistance (μ*L*/*kA*) experienced by the fluid flow also increases, which results in a lower discharge rate (*Q*) for a given pressure drop (Δ*P*). For example, in the case of a flow rate of 0.3 mL/min, we calculate the permeability using Darcy’s law to be ≈1.44 × 10^–12^ and the porosity calculated using the Kozeny–Carman equation was ≈0.63, see Supporting Information, section 3.0. Specifically, the main contribution of the bed height to the rate of flow is observed at pH levels of 6 and 9. The lowest flow rates occur at pH 3 due to particle aggregation. Bed mass variations by a factor of 3 induce minimal response, changing the flow rate from 0.048 to 0.045 mL/min, which is well within the uncertainty of measurement. At pH 6, the flow drops from 0.163 to 0.107 mL/min over the change in bed mass. Similarly, at pH 9, the flow decreases from 0.318 to 0.168 mL/min when increasing the bed mass by the same factor.

Similarly, increasing the electrolyte, NaNO_3_, concentration from 10 to 200 mM (Figure 8b) decreases flow rates, with the largest reduction in flow rates taking place at pH 9 (0.32 to 0.16 mL/min), followed by pH 6 (0.16 to 0.13 mL/min), and by pH 3 (0.045 to 0.034 mL/min). This results from double-layer screening, which reduces electrostatic repulsion and promotes particle coagulation and increased bed packing density^26,29,53^ that hinders the flow rate therein.

**Figure 8:**
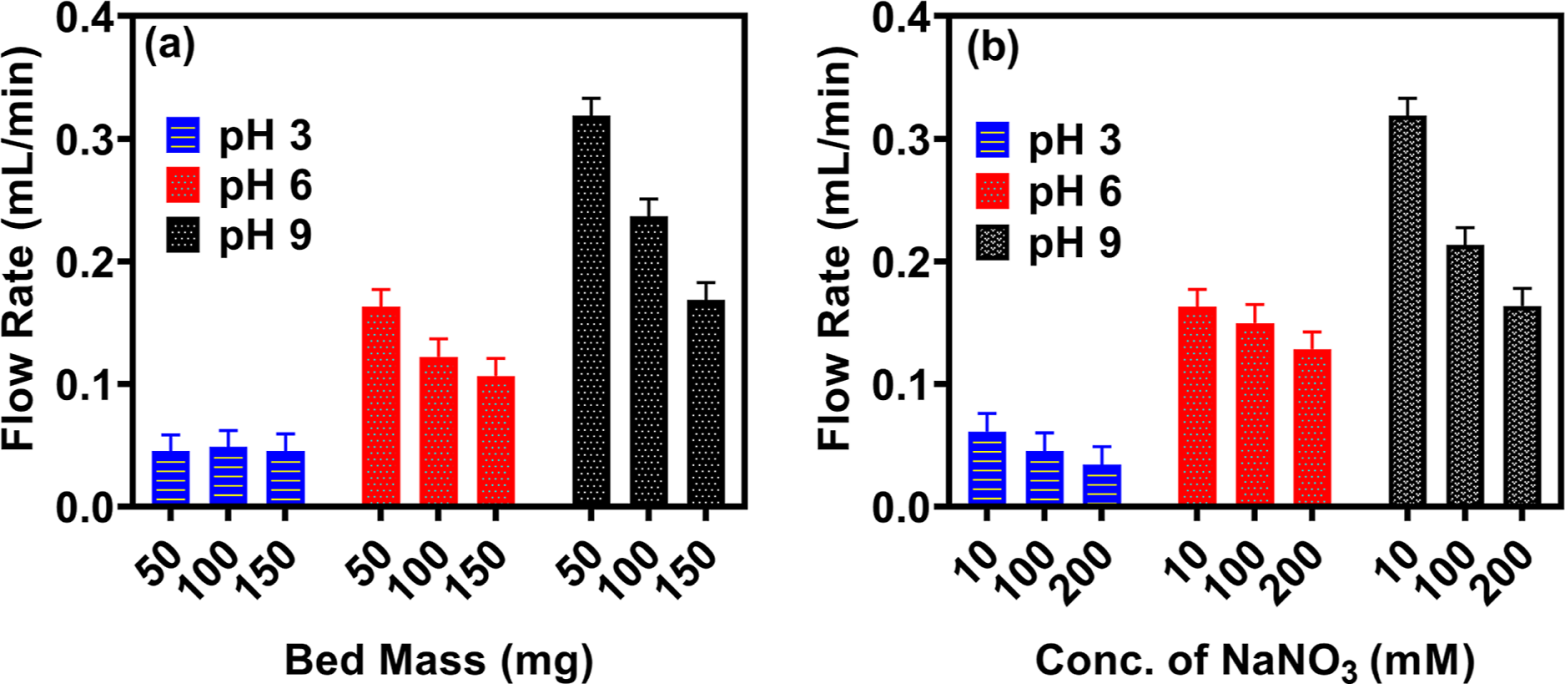
Flow rate variations through packed beds of *h*-PAA-Si particles as a function of (a) bed mass (proportional to bed height) and (b) ionic strength (NaNO_3_ concentration) at different pH levels. Error bars represent standard deviations from triplicate measurements.

## 4 Conclusion

We investigate the behavior of stimuli-responsive silica microparticles functionalized with polymer brushes in packed beds as pH-tunable “colloidal gates” capable of regulating liquid transport. By conjugating poly­(acrylic acid) to aminosilane-grafted silica through variations in the surface amine density, we regulate the pH-dependent swelling/compaction of the materials, mirroring natural clay materials. This allows us leverage surface chemistry to encode the particulate bed permeability. Confined within a packed bed flow column, the modified particles exhibit coagulation/dispersion modulated by solution acidity shifts. Zeta potential and DLS analyses validate reversible aggregation states at both high and low pH. Quantitative permeation measurements reveal transport modulation through adjustable packing rearrangements, which are driven by the interaction of the particle surface chemistry with the pH and ion levels in the solvent.

Permeabilities through the particulate bed columns correlate with protonation state-dependent interactions at the particle surfaces. The pH variations and the permeability are maximized in the case of the densely functionalized *h*-PAA-Si system. Moreover, transport tuning capacity increases with increasing brush densities. However, ionic strength variations dampen variability in the particulate bed permeability due to electrostatic screening of surface charges.

The pH-programmable assembly on the particle surface provides a synthetic platform for actively governing solution flow on demand by colloidal particles using intermolecular interactions. Potential applications include filtration, where the colloidal gate modulates bulk fluid flow (0.04–0.32 mL/min) in aqueous 10 mM NaNO_3_ solutions (pH 2–10) by tuning pH to adjust particle packing, suitable for wastewater treatment. While the current system focuses on bulk flow, future work could explore selective transport of specific molecules (e.g., heavy metals, biomolecules) for separation and delivery applications, building on our reversible pH response and high grafting density.

## Supplementary Material



## Notes

Thermogravimetric analysis of APTES-grafted and PAA-conjugated silica particles. Reversibility of pH responsiveness of *h*-PAA-Si sample: autocorrelation function after reversibility test at pH 3. Measured zeta potential of hydrolyzed silica support from pH 3–10. Optical microscopy images tracking in situ particulate aggregation behaviors modulated by pH. (20× magnification, 100 μm × 100 μm frame) to visualize pH-responsive aggregation kinetics within the suspension. Optical microscopy images of particulate aggregation behaviors modulated by pH after solvent evaporation. (20× magnification, 300 μm × 300 μm frame). Liquid flow rates were measured as a function of packed bed height for 50, 100, and 150 mg. Flow variations from adjusting background salt concentration (NaNO_3_) across different pH values (PDF)

## References

[1] Aksu I., Bazilevskaya E., Karpyn Z. (2015). Swelling of clay minerals in unconsolidated
porous media and its impact on permeability. GeoResJ..

[2] Chang P.-H., Li Z., Jiang W.-T., Jean J.-S. (2009). Adsorption and intercalation of tetracycline
by swelling clay minerals. Appl. Clay Sci..

[3] Alberts, B. ; Johnson, A. ; Lewis, J. ; Raff, M. ; Roberts, K. ; Walter, P. Molecular Biologyof the Cell, 4 th ed.; Garland Sci., 2002.

[4] Wang Z., Li Q., Van Der Bruggen B., Jiang Z., Cahill D. M., Li J., Zhao S. (2025). Photoresponsive Gas-Permeable Membranes: Fundamentals,
Innovations, and Prospects. Adv. Funct. Mater..

[5] Rehmat S., Rizvi N. B., Khan S. U., Ghaffar A., Islam A., Khan R. U., Mehmood A., Butt H., Rizwan M. (2022). Novel Stimuli-Responsive
Pectin-PVP-Functionalized Clay Based Smart Hydrogels for Drug Delivery
and Controlled Release Application. Front. Mater..

[6] Li Z., Zhu Y., Matson J. B. (2022). pH-Responsive Self-Assembling Peptide-Based
Biomaterials:
Designs and Applications. ACS Appl. Bio Mater..

[7] Khaskia M., Shpasser D., Cohen R., Yehezkeli O., Manor O., Gazit O. M. (2022). First-Principle Colloidal Gate for
Controlling Liquid and Molecule Flow Using 2D Claylike Nanoparticles. ACS Appl. Mater. Interfaces.

[8] Prado-Rubio O., Jørgensen S., Jonsson G. (2012). pH control structure design for a
periodically operated membrane separation process. Comput. Chem. Eng..

[9] Kohay H., Gazit O. M. (2024). Regulating adsorption-desorption
through controlled
modification of the 2D pores in layered double hydroxides. Surf. Interfaces..

[10] Niu X., Xu X., Li X., Pan J., Qiu F., Zhao H., Lan M. (2018). Surface charge engineering
of nanosized CuS *via* acidic
amino acid modification enables high peroxidase-mimicking activity
at neutral pH for one-pot detection of glucose. Chem. Commun..

[11] Vangelatos Z., Gu G. X., Grigoropoulos C. P. (2019). Architected
metamaterials with tailored
3D buckling mechanisms at the microscale. EML.

[12] Li Y., Dekel D. R., Manor O. (2021). Surface Acoustic Wave Mitigation
of Precipitate Deposition on a Solid Surface. An Active Self-Cleaning
Strategy. ACS Appl. Mater. Interfaces.

[13] Zhang L., Zhang S., Floer C., Kantubuktha S. A. R., Velasco M. J. G. R., Friend J. (2024). Surface Acoustic Wave-Driven
Enhancement of Enzyme-Linked Immunosorbent Assays: ELISAW. Anal. Chem..

[14] Schacher F., Ulbricht M., Müller A. H. E. (2009). Self-Supporting,
Double Stimuli-Responsive
Porous Membranes From Polystyrene- *block* -poly­(*N*, *N* -dimethylaminoethyl methacrylate)
Diblock Copolymers. Adv. Funct. Mater..

[15] Moreno S., Hübner H., Effenberg C., Boye S., Ramuglia A., Schmitt D., Voit B., Weidinger I. M., Gallei M., Appelhans D. (2022). Redox- and
pH-Responsive Polymersomes
with Ferrocene Moieties Exhibiting Peroxidase-like, Chemoenzymatic
Activity and H_2_ O_2_ -Responsive Release Behavior. Biomacromolecules.

[16] Clodt J. I., Filiz V., Rangou S., Buhr K., Abetz C., Höche D., Hahn J., Jung A., Abetz V. (2013). Double Stimuli-Responsive
Isoporous Membranes via Post-Modification of pH-Sensitive Self-Assembled
Diblock Copolymer Membranes. Adv. Funct. Mater..

[17] Khabibullin, A. ; Zharov, I. In Intelligent Stimuli-Responsive Materials, 1st ed.; Li, Q. , Ed., Wiley, 2013; pp 265–291.

[18] Bohaty A. K., Smith J. J., Zharov I. (2009). Free-Standing
Silica Colloidal Nanoporous
Membranes. Langmuir.

[19] Zharov I., Khabibullin A. (2014). Surface-Modified Silica Colloidal Crystals: Nanoporous
Films and Membranes with Controlled Ionic and Molecular Transport. Acc. Chem. Res..

[20] Huckabee A. G., Yerneni C., Jacobson R. E., Alzate E. J., Chen T.-H., Wirth M. J. (2017). In-column bonded
phase polymerization for improved
packing uniformity. J. Sep. Sci..

[21] Luzinov I., Julthongpiput D., Liebmann-Vinson A., Cregger T., Foster M. D., Tsukruk V. V. (2000). Epoxy-Terminated
Self-Assembled Monolayers: Molecular
Glues for Polymer Layers. Langmuir.

[22] Caruso F., Furlong D. N., Ariga K., Ichinose I., Kunitake T. (1998). Characterization
of Polyelectrolyte Protein Multilayer Films by Atomic Force Microscopy,
Scanning Electron Microscopy, and Fourier Transform Infrared Reflection
Absorption Spectroscopy. Langmuir.

[23] Sankhala K., Koll J., Abetz V. (2020). Facilitated Structure Formation in
Isoporous Block Copolymer Membranes upon Controlled Evaporation by
Gas Flow. Membranes.

[24] Eygeris Y., Ulery N., Zharov I. (2023). pH-Responsive
Membranes from Self-Assembly
of Poly­(2-(dimethylamino)­ethyl methacrylate) Brush Silica Nanoparticles. Langmuir.

[25] Kozlovskaya V., Kharlampieva E., Mansfield M. L., Sukhishvili S. A. (2006). Poly­(methacrylic
acid) Hydrogel Films and Capsules: Response to pH and Ionic Strength,
and Encapsulation of Macromolecules. Chem. Mater..

[26] Homede E., Abo Jabal M., Manor O. (2020). Connecting Surface-Forces-Based Energy
Barriers to Nonhomogeneous Colloidal Structures to Appear from Volatile
Binary Mixtures of Same Size Nanoparticle Species. Adv. Funct. Mater..

[27] Onuh G., Harries D., Manor O. (2024). Depletion-Induced
Self-Assembly of
Colloidal Particles on a Solid Substrate. Langmuir.

[28] Zhao C., Zhang W., Van Den
Ende D., Mugele F. (2020). Electroviscous effects
on the squeezing flow of thin electrolyte solution films. J. Fluid Mech..

[29] Onuh G., Bar-On R., Manor O. (2023). Particle Network
Self-Assembly of
Similar Size Sub-Micron Calcium Alginate and Polystyrene Particles
Atop Glass. Macromol. Biosci..

[30] Semenova A., Giles L. W., Vidallon M. L. P., Follink B., Brown P. L., Tabor R. F. (2023). The structure of
colloidal polyethylenimine–silica
nanocomposite microparticles. Particuology.

[31] Rosenholm J. B. (2016). On the
incompatibilities of interaction scales and processes with focus on
the work of adhesion. Adv. Colloid Interface
Sci..

[32] Sgouros A. P., Revelas C. J., Lakkas A. T., Theodorou D. N. (2021). Potential
of Mean Force between Bare or Grafted Silica/Polystyrene Surfaces
from Self-Consistent Field Theory. Polymers.

[33] Dai J., Bao Z., Sun L., Hong S. U., Baker G. L., Bruening M. L. (2006). High-Capacity
Binding of Proteins by Poly­(Acrylic Acid) Brushes and Their Derivatives. Langmuir.

[34] Zhang B.-b., Chen X.-j., Fan X.-d., Zhu J.-j., Wei Y.-h., Zheng H.-s., Zheng H.-y., Wang B.-h., Piao J.-g., Li F.-z. (2021). Lipid/PAA-coated
mesoporous silica nanoparticles for dual-pH-responsive
codelivery of arsenic trioxide/paclitaxel against breast cancer cells. Acta Pharmacol. Sin..

[35] Sypabekova M., Hagemann A., Rho D., Kim S. (2023). Review: 3-Aminopropyltriethoxysilane
(APTES) Deposition Methods on Oxide Surfaces in Solution and Vapor
Phases for Biosensing Applications. Biosensors.

[36] Swift T., Swanson L., Geoghegan M., Rimmer S. (2016). The pH-responsive behaviour
of poly­(acrylic acid) in aqueous solution is dependent on molar mass. Soft Matter.

[37] Lim V. T., Bayly C. I., Fusti-Molnar L., Mobley D. L. (2019). Assessing the Conformational
Equilibrium of Carboxylic Acid via Quantum Mechanical and Molecular
Dynamics Studies on Acetic Acid. J. Chem. Inf.
Model..

[38] Coelho L. H., Gutz I. G. (2006). Trace analysis of
acids and bases by conductometric
titration with multiparametric non-linear regression. Talanta.

[39] Gazit O. M., Katz A. (2012). Grafted Poly­(1, 4-beta-glucan)
Strands on Silica: A Comparative Study
of Surface Reactivity as a Function of Grafting Density. Langmuir.

[40] Brittain W. J., Minko S. (2007). A structural definition
of polymer brushes. J. Polym. Sci. A Polym.
Chem..

[41] Merzougui C. E., Bacchin P., Aimar P., Causserand C., Roblin P. (2025). Analysis of HSA–PAA Complexation
Using SEC-SAXS
Combination: Unraveling Stoichiometry, Reversibility, and Interaction
Specificity. Biomacromolecules.

[42] Yang R., Wang F., Blunk R. H., Angelopoulos A. P. (2010). Competing
effects of silanol surface concentration and solvent dielectric constant
on electrostatic layer-by-layer assembly of silica nanoparticles on
gold. J. Colloid Interface Sci..

[43] Shibayama M., Karino T., Okabe S. (2006). Distribution
analyses of multi-modal
dynamic light scattering data. Polymer.

[44] Chachanidze R., Xie K., Massaad H., Roux D., Leonetti M., De Loubens C. (2022). Structural
characterization of the interfacial self-assembly of chitosan with
oppositely charged surfactant. J. Colloid Interface
Sci..

[45] Alexander M., Dalgleish D. G. (2006). Dynamic
Light Scattering Techniques and Their Applications
in Food Science. Food Biophysics.

[46] Ong G. M., Gallegos A., Wu J. (2020). Modeling Surface Charge
Regulation
of Colloidal Particles in Aqueous Solutions. Langmuir.

[47] O’Shaughnessy B., Vavylonis D. (2003). Irreversible
adsorption from dilute polymer solutions. Eur.
Phys. J. E.

[48] Adamczyk Z., Bratek A., Jachimska B., Jasinski T., Warszynski P. (2006). Structure
of Poly­(acrylic acid) in Electrolyte Solutions Determined from Simulations
and Viscosity Measurements. J. Phys. Chem. B.

[49] Gu Y., Li D. (2000). The zeta-Potential of Glass Surface in Contact with Aqueous Solutions. J. Colloid Interface Sci..

[50] Olhero S., Ferreira J. (2004). Influence of particle size distribution on rheology
and particle packing of silica-based suspensions. Powder Technol..

[51] Dishon M., Zohar O., Sivan U. (2009). From Repulsion
to Attraction and
Back to Repulsion: The Effect of NaCl, KCl, and CsCl on the Force
between Silica Surfaces in Aqueous Solution. Langmuir.

[52] Armstrong R. T., McClure J. E., Berrill M. A., Rücker M., Schlüter S., Berg S. (2016). Beyond Darcy’s
law: The role
of phase topology and ganglion dynamics for two-fluid flow. Phys. Rev. E.

[53] Homede E., Zigelman A., Abezgauz L., Manor O. (2018). Signatures of van der
Waals and Electrostatic Forces in the Deposition of Nanoparticle Assemblies. J. Phys. Chem. Lett..

