# Supplementary material for: Stimuli-Responsive Colloidal Gate for Active Modulation of Fluid Flow in Packed Beds
Source: Langmuir. 2025 Sep 16;41(38):26202–12. doi: 10.1021/acs.langmuir.5c03082 (PMC12490015; doi:10.1021/acs.langmuir.5c03082)
Supplement: Supplementary file 1 [file la5c03082_si_001.pdf]

Supporting Information

Stimuli-Responsive Colloidal Gate for Active Modulation of Fluid Flow in Packed Beds

Gideon Onuh,† Ronit Bitton,‡ Oz M. Gazit,*,† and Ofer Manor*,†

†The Wolfson Department of Chemical Engineering, Technion - Israel Institute of Technology, Haifa 3200000, Israel

‡Department of Chemical Engineering, Ben-Gurion University of the Negev, Beer-Sheva 84105, Israel

E-mail: ozg@technion.ac.il; manoro@technion.ac.il

Number of pages: 13

Number of figures: 12

Number of schemes:1

Number of tables: 1

Table of Contents

Stimuli-Responsive Colloidal Gate for Active Modulation of Fluid Flow in Packed Beds S1

1.0 Back Titration S2

2.0 Calculation of Particle Diameters Using Autocorrelation Function S10

3.0 Permeability Measurement S11

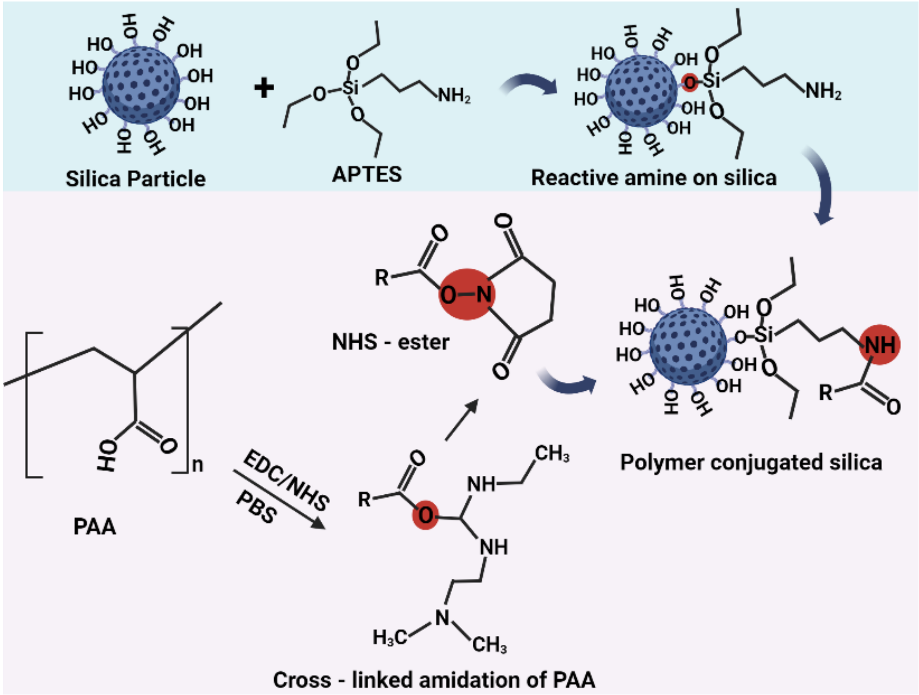

Figure S1: Schematic of the sol-gel reaction process for silica nanoparticle functionalization with aminopropyltriethoxysilane (APTES) via condensation and subsequent poly(acrylic acid) (PAA) conjugation to surface amine groups through carbodiimide coupling chemistry.

1.0 Back Titration

Aminopropylsilane (APTES) grafting density was determined by acid-base back titration. Silica particles (1 g) bearing surface amine moieties were suspended in 25 mL of 0.1 M HCl and vigorously stirred for 10 min. This protonated surface amines to ammonium chloride quantitatively. The suspension was then centrifuged, and the supernatant was carefully isolated, representing unreacted HCl. This aliquot was titrated against 0.1 M NaOH to the equivalence point, registering as the volume (V_2) of the base consumed. The volume of HCl reacted (V_1 - V_2) was equivalent to millimoles of surface amino groups protonated on the silica, given the acid concentration. Dividing this value by the particle mass yielded the grafting density as millimoles of aminopropyl groups per gram of support.

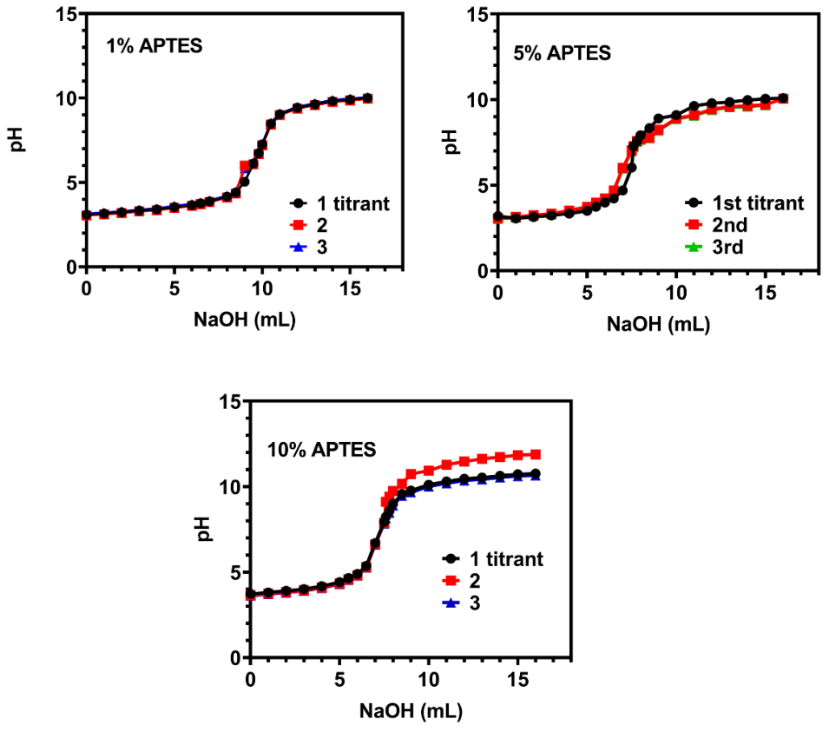

Figure S2: Titrimetric curves for silica support with 1, 5 and 10 % APTES concentrations

Table S1: Table of equipment and operating conditions

Method	Instrument	Settings	Software
DRIFTS	Bruker Tensor ll	Wavenumber range: 400-4000 cm^{-1}: Resolution: 4 cm^{-1}, Scans: 64; Sample mixed with KBr (1:100 w/w); Diffuse reflectance accessory at 25°C	OPUS 7.5 for data collection and spectral analysis
SAXS	Anton Paar SAXSpoint 2.0	Cu Kα radiation (λ = 1.5418 Å); q-range: 0.01-0.4 $Å^{-1}$; Exposure time: 600 s; Sample in 1 mm quartz capillary with 10 mg/mL suspension in 10 mM $NaNO_3$: (pH 9)	SAXS analysis for data fitting and layer thickness calculation
TGA	Setaram Insstrument (Kep Technologi)	Temperature range: 25-800 °C; Heating rate: 10 °C/min; Sample mass: 5-10 mg; Argon 75% /Air 25% atmosphere	Calisto acquisition for thermogram processing and mass loss calculation
DLS	Malvern Zetasizer Nano ZS	Scattering angle: 173° (backscatter); Wavelength: 633 nm (He-Ne laser); Sample: 0.1 mg/mL in 10 mM $NaNO_3$: (pH 2-10); Measurement at 25 °C; Triplicate runs	Zetasizer Software 7.13 for size distribution analysis
Zeta Potential	Malvern Zetasizer Nano ZS	Electrophoretic mobility measured in 10 mM $NaNO_3$: (pH 2-10); Sample: 0.1 mg/mL; Disposable folded capillary cell (DTS1070); Measurement at 25 °C; Triplicate runs	Zetasizer Software 7.13 for zeta potential calculation using Smoluchowski model

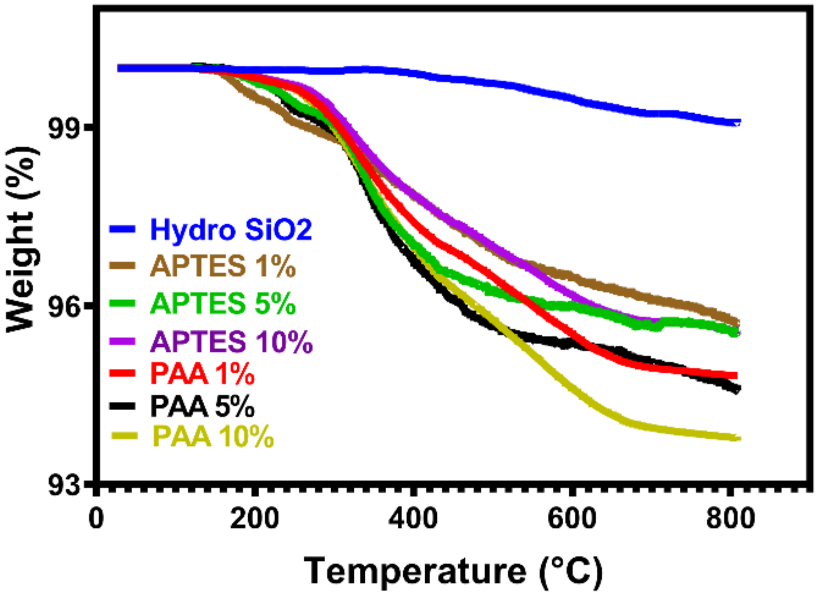

Figure S3: Thermogravimetric analysis of APTES-grafted and PAA-conjugated silica particles

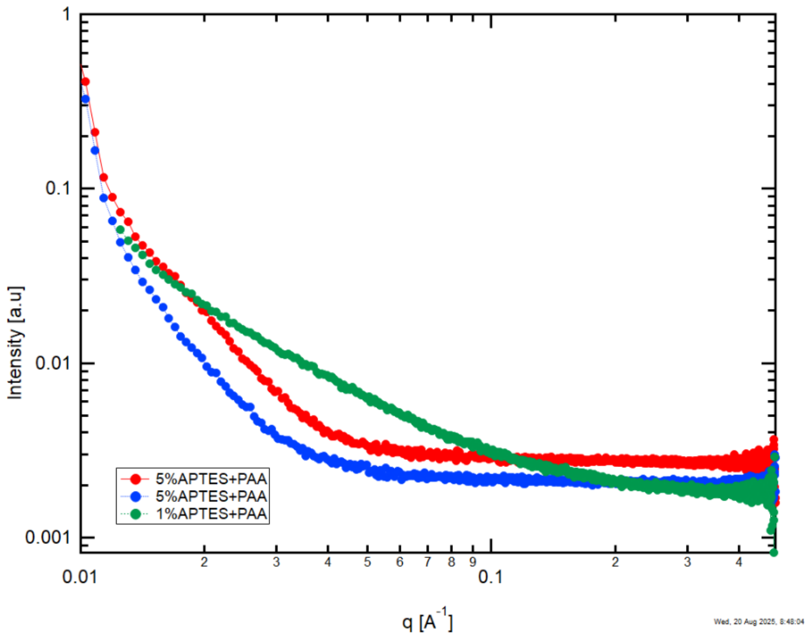

Fig S4: Show that even without solvent subtraction, the similarity between the SAXS curves at 5% and 10%, as well as their clear difference from the 1% curve, is evident

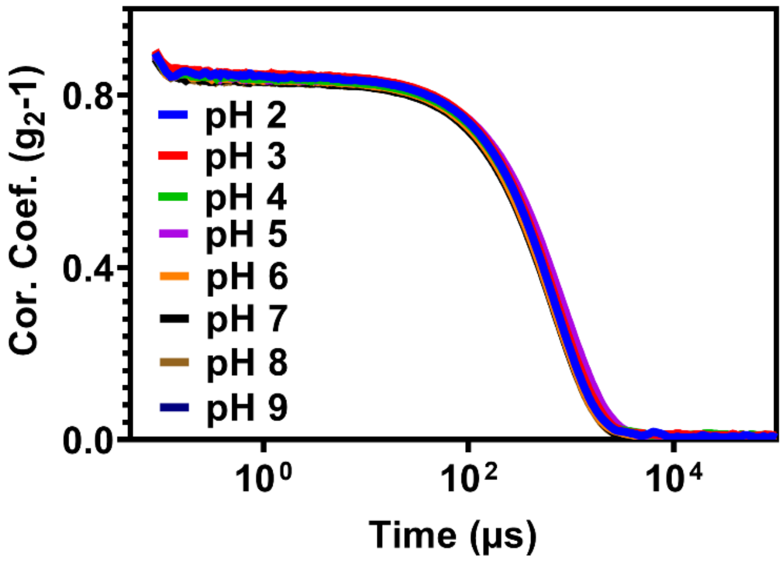

Figure S5: Reversibility of pH responsiveness of 10% PAA-Si sample: autocorrelation function after reversibility test at pH 3.

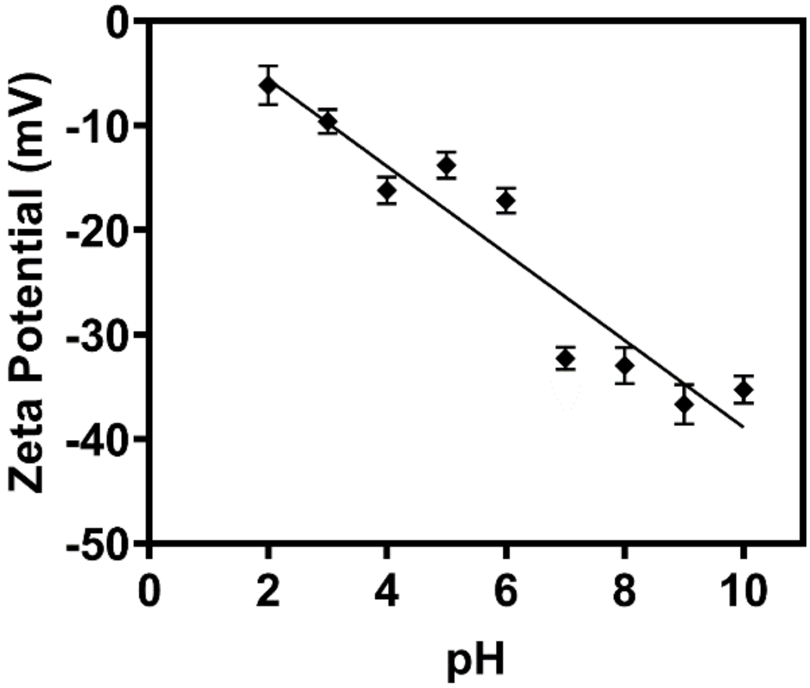

Figure S6: Measured zeta potential of hydrolyzed silica support from pH 3–10.

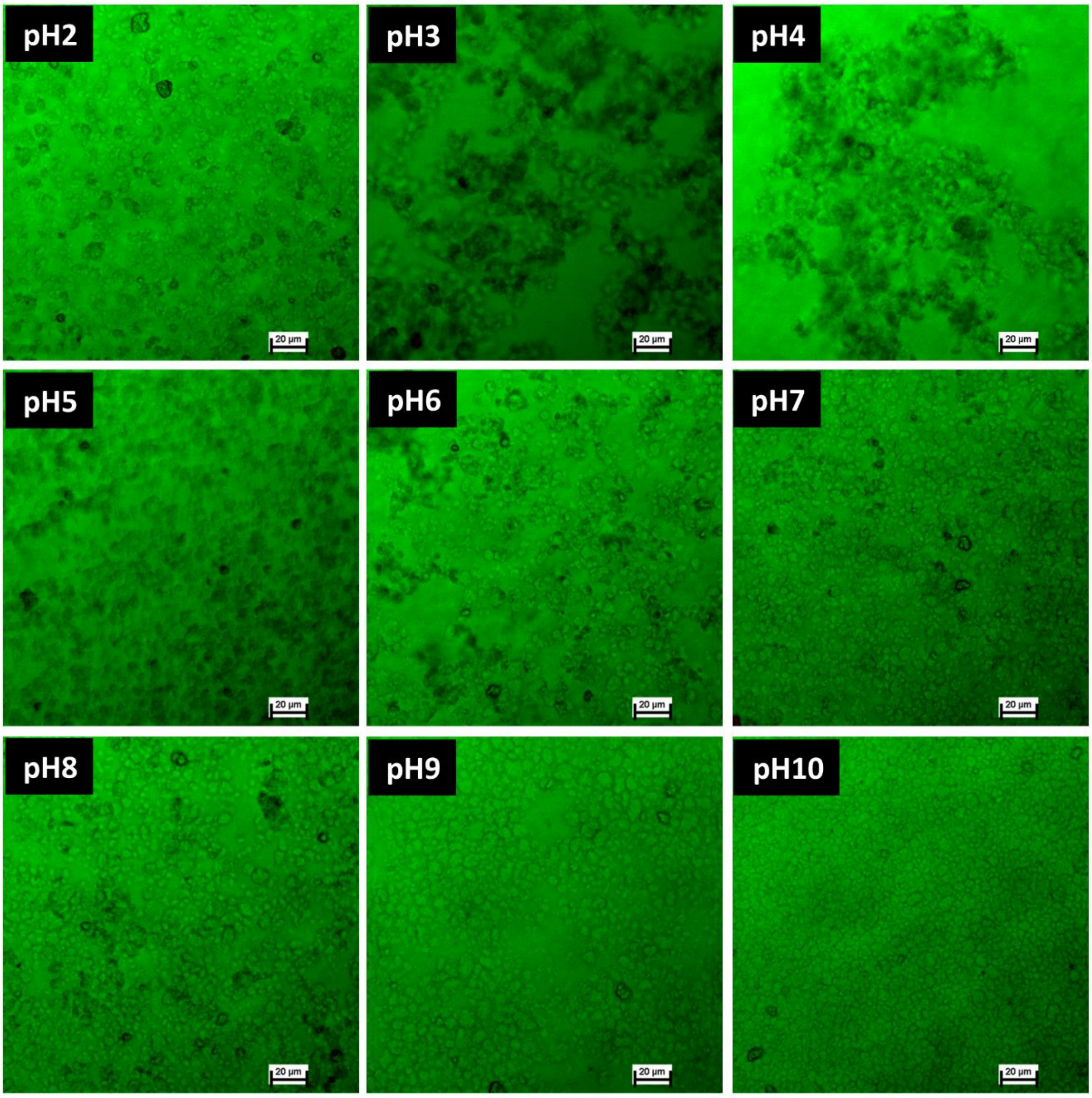

Figure S7: Optical microscopy images tracking in situ particulate aggregation behaviours modulated by pH. (20X magnification, 100 µm × 100 µm frame) to visualize pH-responsive aggregation kinetics within the suspension.

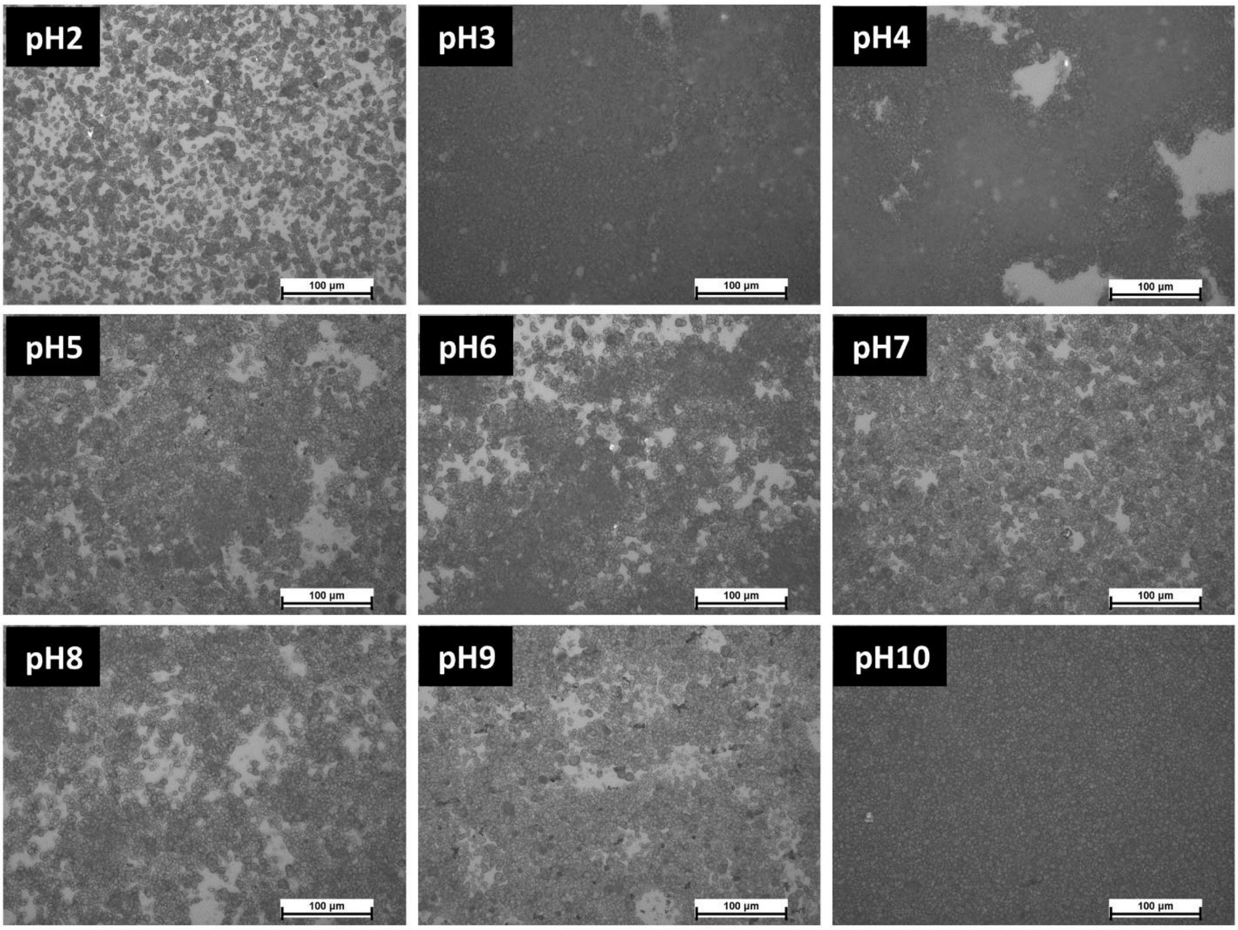

Figure S8: Optical microscopy images of particulate aggregation behaviours modulated by pH after solvent evaporation. (20X magnification, 300 µm × 300 µm frame).

Name	Mean	Standard Deviation	RSD	Minimum	Maximum
Z-Average (nm)	6588	-	-	6588	6588
Polydispersity Index (PI)	0.1728	-	-	0.1728	0.1728
Peak 1 Mean by Intensity ordered by area (nm)	5468	-	-	5468	5468
Peak 1 Area by Intensity ordered by area (%)	100	-	-	100	100

Name	Mean	Standard Deviation	RSD	Minimum	Maximum
Z-Average (nm)	7305	-	-	7305	7305
Polydispersity Index (PI)	1	-	-	1	1
Peak 1 Mean by Intensity ordered by area (nm)	4823	-	-	4823	4823
Peak 1 Area by Intensity ordered by area (%)	100	-	-	100	100

Name	Mean	Standard Deviation	RSD	Minimum	Maximum
Z-Average (nm)	5518	-	-	5518	5518
Polydispersity Index (PI)	0.1406	-	-	0.1406	0.1406
Peak 1 Mean by Intensity ordered by area (nm)	5270	-	-	5270	5270
Peak 1 Area by Intensity ordered by area (%)	100	-	-	100	100

Name	Mean	Standard Deviation	RSD	Minimum	Maximum
Z-Average (nm)	9068	-	-	9068	9068
Polydispersity Index (PI)	1	-	-	1	1
Peak 1 Mean by Intensity ordered by area (nm)	4821	-	-	4821	4821
Peak 1 Area by Intensity ordered by area (%)	100	-	-	100	100

Name	Mean	Standard Deviation	RSD	Minimum	Maximum
Z-Average (nm)	7924	-	-	7924	7924
Polydispersity Index (PI)	1	-	-	1	1
Peak 1 Mean by Intensity ordered by area (nm)	4863	-	-	4863	4863
Peak 1 Area by Intensity ordered by area (%)	100	-	-	100	100

Name	Mean	Standard Deviation	RSD	Minimum	Maximum
Z-Average (nm)	6099	-	-	6099	6099
Polydispersity Index (PI)	0.5058	-	-	0.5058	0.5058
Peak 1 Mean by Intensity ordered by area (nm)	5325	-	-	5325	5325
Peak 1 Area by Intensity ordered by area (%)	100	-	-	100	100

Figure S9: Reversibility of pH responsiveness of *h*-PAA-Si sample. Dh of aggregates measured using DLS by alternating pH of the suspension between 4.

Name	Mean	Standard Deviation	RSD	Minimum	Maximum
Z-Average (nm)	4866	-	-	4866	4866
Polydispersity Index (PI)	1	-	-	1	1
Peak 1 Mean by Intensity ordered by area (nm)	3836	-	-	3836	3836
Peak 1 Area by Intensity ordered by area (%)	100	-	-	100	100

Name	Mean	Standard Deviation	RSD	Minimum	Maximum
Z-Average (nm)	3502	-	-	3502	3502
Polydispersity Index (PI)	0.2185	-	-	0.2185	0.2185
Peak 1 Mean by Intensity ordered by area (nm)	3995	-	-	3995	3995
Peak 1 Area by Intensity ordered by area (%)	100	-	-	100	100

Name	Mean	Standard Deviation	RSD	Minimum	Maximum
Z-Average (nm)	4195	-	-	4195	4195
Polydispersity Index (PI)	1	-	-	1	1
Peak 1 Mean by Intensity ordered by area (nm)	934	-	-	934	934
Peak 1 Area by Intensity ordered by area (%)	100	-	-	100	100

Name	Mean	Standard Deviation	RSD	Minimum	Maximum
Z-Average (nm)	4178	-	-	4178	4178
Polydispersity Index (PI)	0.07583	-	-	0.07583	0.07583
Peak 1 Mean by Intensity ordered by area (nm)	4476	-	-	4476	4476
Peak 1 Area by Intensity ordered by area (%)	100	-	-	100	100

Name	Mean	Standard Deviation	RSD	Minimum	Maximum
Z-Average (nm)	4948	-	-	4948	4948
Polydispersity Index (PI)	0.1921	-	-	0.1921	0.1921
Peak 1 Mean by Intensity ordered by area (nm)	5190	-	-	5190	5190
Peak 1 Area by Intensity ordered by area (%)	100	-	-	100	100

Figure S10: Reversibility of pH responsiveness of *h*-PAA-Si sample. Dh of aggregates measured using DLS by alternating pH of the suspension between 9.

2.0 Calculation of Particle Diameters Using Autocorrelation Function

DLS measures the time-dependent fluctuations in light scattered by particles undergoing Brownian motion. The autocorrelation function, G(τ), describes how the scattered light intensity correlates over time (τ), reflecting the particle motion. The decay rate of this function is related to the particle's diffusion coefficient, which is used to calculate the hydrodynamic diameter.

a. Obtain the Autocorrelation Function:

DLS instruments provide the intensity autocorrelation function, typically denoted as $G^{(2)}(\tau)$, where: $G^{(2)}(\tau) = \langle I(t)I(t+\tau)\rangle / \langle I(t)\rangle^2$

Here, I(t) is the scattered light intensity at time t, and τ is the delay time.

b. Extract the decay rate (Γ):

Where $g^{(1)}(\tau) = \exp(-\Gamma\tau)$

$g^{(1)}(\tau) = \sqrt{(G^{(2)}(\tau) - 1)/\beta}$ is the electric field autocorrelation function.

The decay rate (Γ) is related to the diffusion coefficient as $\Gamma = Dq^2$

Where the scattering vector $q = \frac{4\pi n}{\lambda}\sin(\frac{\theta}{2})$

Where; η= is the refractive index of the medium

λ = wavelength if the laser in a vacuum

θ = scattering angle

so, the diffusion coefficient $D = \frac{\Gamma}{q^2}$ (λ = 633 nm, η = 1.33, θ = 173^0)

Then $q = \frac{4\pi .1.33}{633.10^{-9}m}\sin(\frac{173}{3}) = 1.87.10^7 m^{-1}$

c. Apply the Stokes-Einstein Equation;
The hydrodynamic diameter d_H is calculated as $d_H = \frac{k_B T}{3\pi \eta D}$

k_B: Boltzmann constant ($1.38.10^{-23}$ J/K)

T: absolute temperature (298 K)

η: viscosity of water 0.0887 mPa.s)

3.0 Permeability Measurement

We calculate the permeability of the bed using Darcy's Law and the porosity using the Kozeny – Carman equation.

Parameters:

Column volume: 2 mL (0.002 L = 0.002 . 10^{-3} m^3),

Column diameter: 2 cm (0.02 m),

Flow rate (Q): 0.3 mL/min (0.3 . 10^{-6} m^3/60 s = 5 . 10^{-9} m^3/s),

Particle diameter: 4 µm (4 . 10^{-6} m).

Step 1: Calculate Permeability Using Darcy's Law

$$Q = \frac{kA\Delta P}{\mu L}$$

Where:

Q: Flow rate (m^3/s),

K: Permeability (m^2),

A: Cross-sectional area of the column (m^2),

ΔP: Pressure drop across the column (Pa),

µ: Dynamic viscosity of the fluid (Pa·s),

L: height of the liquid (m).

If Q = 0.3 mL/min = 5 . 10^{-9} m^3/s

Column diameter = 0.02 m, so radius (r = 0.01 m),

Cross-sectional area A = $\pi r^2 = \pi(0.01\ m)^2$ = 3.1416 . 10^{-4} m^2

Length (L) = V/A =

$$Length\ (L) = \frac{V}{A} = \frac{0.002\ .\ 10^{-3}\ \mathrm{m}^3}{3.1416\ .\ 10^{-4}-4\ \mathrm{m}^2} = 0.00637\ m$$

Viscosity of water at 25 °C, µ = 0.89 . 10^{-3} Pa·s = (0.89 mPa·s).

Pressure drop ΔP = $\rho_{1} - \rho_{0}$ = ρgh = (1000 kg/m^3 . 10 m/s^2 . 0.00637 m) = 63.7 Pa or (Kg/ms^2)

- We assumed the sphericity of the particles in the packed bed (ϕ_S= 1.0 for spherical particles) [1][2].

The permeability according to Darcy's law:

$$k = \frac{Q\mu L}{A\Delta P}$$

$$k = \frac{\left(5\,.\,10^{-9}\,\frac{\mathrm{m}^3}{\mathrm{s}}\right).\ \left(0.89\,.\,10^{-3}\,\frac{Kg}{ms^2}\quad \mathrm{s}\right).\,(0.00637\ \mathrm{m})}{(3.1416\,.\,10^{-4}\,m^2)\,.\ (63.7\ \mathrm{Kg}/\ ms^2)} = 1.44\,.\ 10^{-12}\ m^2$$

The Porosity using Kozeny – Carman equation

We calculate the porosity of the bed using the Kozeny - Carman equation which relates the permeability, particle size and bed structure.

$$k = \frac{\epsilon^3 d_p^2}{180(1-\epsilon)^2}$$

Where:

K = Permeability (m^2)

ϵ = Porosity (dimensionless),

d_p = Particle diameter (4×10^{-6} m),

180: Kozeny constant for spherical particles (commonly used for packed beds)

By solving this non-linear equation, we estimate the bed porosity to be 0.63 in our system.

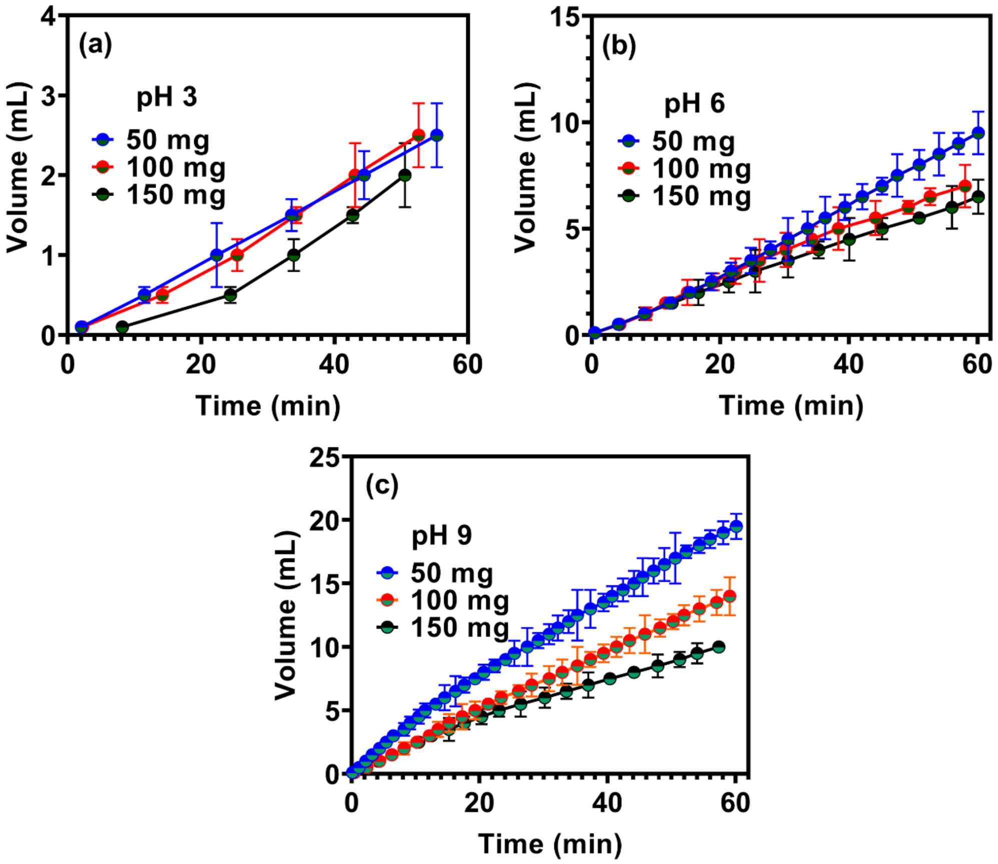

Figure S11: Liquid flow rates were measured as a function of packed bed height for 50, 100, and 150 mg

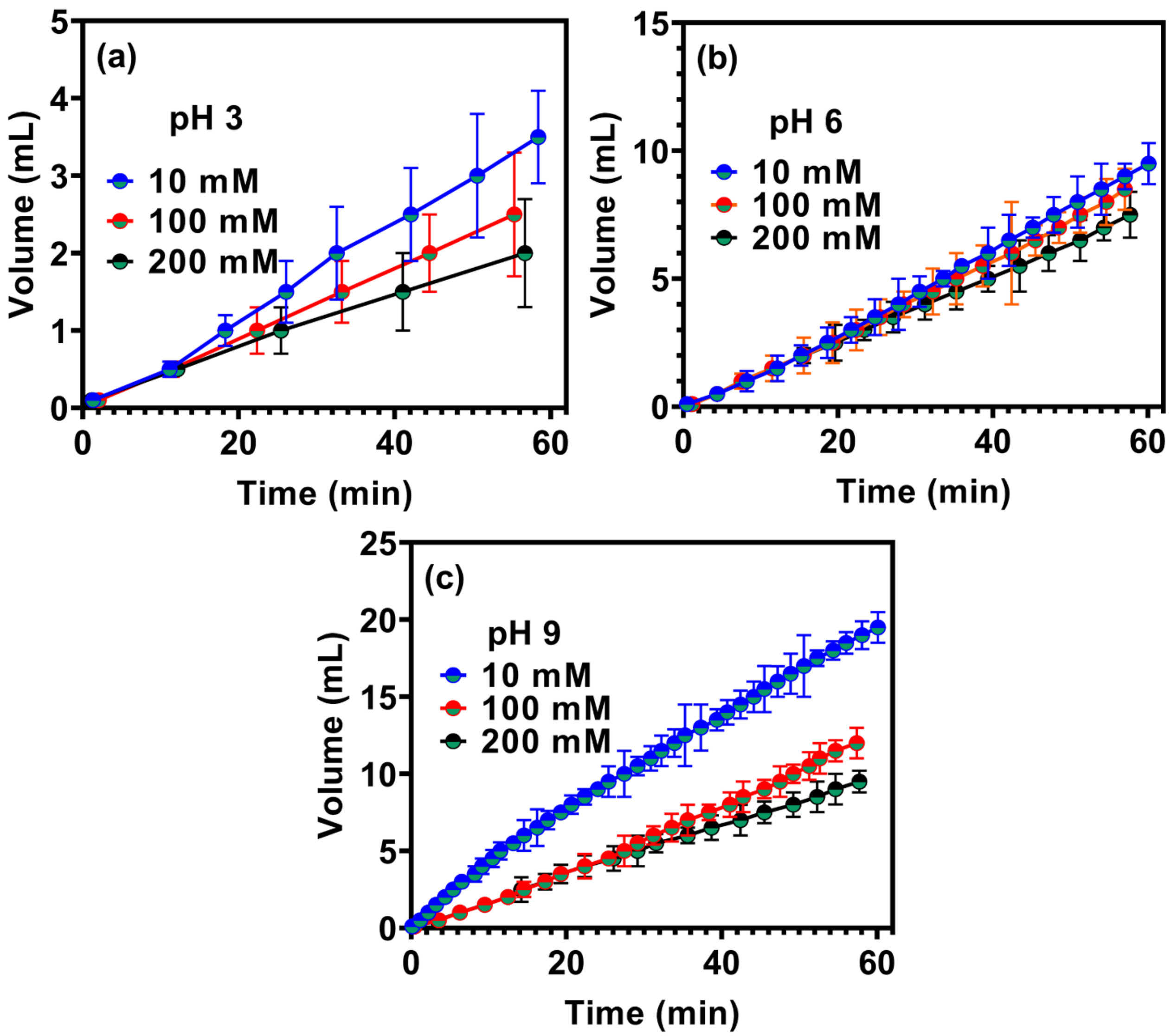

Figure S12: Flow variations from adjusting background salt concentration ($NaNO_3$) across different pH values

References

[1] G.M. Fair, L.P. Hatch, Fundamental factors governing the streamline flow of water through sand, J. AWWA 25 (1933) 1551–1565.

[2] P.C. Carman, "Fluid flow through granular beds." Transactions, Institution of Chemical Engineers, London, 15: 150-166, 1937.
